# The YOUth cohort study: MRI protocol and test-retest reliability in adults

**DOI:** 10.1016/j.dcn.2020.100816

**Published:** 2020-07-08

**Authors:** Elizabeth E.L. Buimer, Pascal Pas, Rachel M. Brouwer, Martijn Froeling, Hans Hoogduin, Alexander Leemans, Peter Luijten, Bastiaan J. van Nierop, Mathijs Raemaekers, Hugo G. Schnack, Jalmar Teeuw, Matthijs Vink, Fredy Visser, Hilleke E. Hulshoff Pol, René C.W. Mandl

**Affiliations:** a UMCU Brain Center, University Medical Center Utrecht, University Utrecht, Utrecht, the Netherlands; b Image Sciences Institute, University Medical Center Utrecht and Utrecht University, Utrecht, the Netherlands; c Department of Radiology, University Medical Center Utrecht, Utrecht, the Netherlands; d Department of Psychology, Utrecht University, Utrecht, the Netherlands; e Philips Healthcare, Best, the Netherlands

**Keywords:** CSF, cerebrospinal fluid, DWI, diffusion-weighted imaging, fMRI, functional magnetic resonance imaging, GM, gray matter, ICC, intraclass correlation coefficient, PD, percentage difference, ROI, region of interest, rs-fMRI, resting-state functional magnetic resonance imaging, sMRI, structural magnetic resonance imaging, SNR, signal-to-noise ratio, SFNR, signal-to-fluctuation-noise ratio, QC, quality control, YOUth cohort, Youth of Utrecht cohort, WM, white matter, Adolescence, Intraclass correlation coefficient, Longitudinal brain development, Magnetic resonance imaging, Test-retest reliability, Youth (Youth of Utrecht) cohort study

## Abstract

The YOUth cohort study is a unique longitudinal study on brain development in the general population. As part of the YOUth study, 2000 children will be included at 8, 9 or 10 years of age and planned to return every three years during adolescence. Magnetic resonance imaging (MRI) brain scans are collected, including structural T1-weighted imaging, diffusion-weighted imaging (DWI), resting-state functional MRI and task-based functional MRI. Here, we provide a comprehensive report of the MR acquisition in YOUth Child & Adolescent including the test-retest reliability of brain measures derived from each type of scan. To measure test-retest reliability, 17 adults were scanned twice with a week between sessions using the full YOUth MRI protocol. Intraclass correlation coefficients were calculated to quantify reliability. Global brain measures derived from structural T1-weighted and DWI scans were reliable. Resting-state functional connectivity was moderately reliable, as well as functional brain measures for both the inhibition task (stop versus go) and the emotion task (face versus house). Our results complement previous studies by presenting reliability results of regional brain measures collected with different MRI modalities. YOUth facilitates data sharing and aims for reliable and high-quality data. Here we show that using the state-of-the art YOUth MRI protocol brain measures can be estimated reliably.

## Highlights

• Test-retest reliability of structural MRI and DWI data was good.
• Test-retest reliability of resting-state and task-related fMRI was moderate.
• Overall, global brain measures are more reliable than local brain measures.

## 1 Introduction

To quantify and understand atypical brain development, we need to first understand typical brain development. In the past two decades multiple longitudinal magnetic resonance imaging (MRI) studies investigating brain development have been initiated around the world (Bjork et al., 2017; Braams et al., 2015; Brown et al., 2015; Evans and Brain Development Cooperative, 2006; Giedd et al., 1999; Herting et al., 2014; Schumann et al., 2010; Tamnes et al., 2013; van Soelen et al., 2012; Wendelken et al., 2017; White et al., 2013; Yap et al., 2011). These cohorts provide rich datasets that can yield important insights on the concept of optimal brain development and individual developmental trajectories.

Studying subtle inter-individual differences in the development of brain structure and function requires reliable brain measures. One way to assess reliability is by using a test-retest design, in which subjects are scanned repeatedly in a short time period. Although, a between-scan interval of a month or less seems appropriate, this data is rarely collected in children and the shortest time intervals found in fMRI test-retest literature are between 3–6 months (Herting et al., 2018). Short time intervals ensure that changes related to plasticity or development are negligible and therefore intra-individual variation between these scan sessions can be regarded as noise. Test-retest reliability can be quantified with the intraclass correlation coefficient (ICC) (Bartko and Carpenter, 1976; McGraw and Wong, 1996; Shrout and Fleiss, 1979), a widely-used statistic in both structural and functional MRI studies.

YOUth (Youth of Utrecht) is an ongoing longitudinal cohort study that comprises two independent cohorts: YOUth Baby & Child and YOUth Child & Adolescent. Together these cohorts should provide a complete overview of development from 20 weeks of gestational age to adolescence. The aim of the YOUth study is to map variation in typical neurocognitive development and investigate why some children develop problematic behavior and others show resilience. To this end, an extensive dataset is collected, including MRI, eye tracking, parent-child observations, computer tasks, cognitive measurements and questionnaires on behavior, personality, health, lifestyle, parenting, child development, use of (social) media and more. Furthermore, blood samples, buccal swabs, saliva and hair samples are collected. More information about the study design and a full overview of the collected data can be found at the website: www.uu.nl/en/research/youth-cohort-study (see also: Onland-Moret et al., 2020 in this issue). The current paper focuses on the MRI data collected in the YOUth Child & Adolescent cohort.

The YOUth MRI protocol comprises different types of MRI scans, i.e. structural T1-weighted images, diffusion-weighted images (DWI), resting-state functional MRI (rs-fMRI) scans and task-based functional MRI (fMRI) scans. YOUth specifically focuses on self-regulation and social competence. Therefore, two fMRI tasks were chosen to match these themes: the inhibition task as a proxy of self-regulation and the emotion task as a proxy of social competence.

YOUth is designed to facilitate data sharing with internal and external researchers guided by the FAIR (Findable, Accessible, Interoperable and Reusable) data principles (Wilkinson et al., 2016). In this paper we provide a transparent report of the collected MRI data. The aim of this paper is two-fold: First, to describe the full YOUth MRI protocol including its state-of-the-art MRI acquisition protocol. Second, to quantify the test-retest reliability of the included MRI acquisitions. To assess test-retest reliability of the YOUth MRI protocol, we included a sample of 17 healthy adult volunteers.

## 2 Material and methods

### 2.1 YOUth child & adolescent

#### 2.1.1 Sample and recruitment

YOUth Child & Adolescent aims to include a total of 2000 children from the general population and their parents or caregivers. Children are recruited mostly at primary schools in the province of Utrecht, the Netherlands. At the first measurement children are 8, 9 or 10 years old. Follow-up measurements are planned every three years during adolescence.

#### 2.1.2 In- and exclusion criteria

All children in the specified age categories can be included as long as they are physically and mentally capable to participate. Furthermore, we exclude children if they or their parents do not master the Dutch language enough to give informed consent or participate in the different subparts of the study. Atypically developing children are not excluded but also not specifically selected. Children that do not meet MR safety criteria (absence of specific metal implants including most braces) were still welcome to participate in the other parts of the study.

### 2.2 The YOUth MRI protocol

#### 2.2.1 Mock procedure

Prior to scanning, children undergo a practice session in a mock scanner. Implementing a mock procedure mimicking the actual experience in the scanner has been shown to decrease scanner-related distress in children (Durston et al., 2009). For YOUth, an older MR scanner model, no longer operational, is reconstructed to be used as a mock scanner to make the experience as authentic as possible. Print-outs of T1-weighted scans with severe motion artefacts and negligible motion artefacts are shown to explain the importance of not moving in the scanner at the level of the child. During the simulation, children are positioned in a mock scanner with headphones on. To familiarize them to the noise of the different MRI sequences sound recordings of these sequences are played, while they practice the inhibition task that they will perform in the real scanner. Following the scanner simulation, the child, the parent or guardian and the research assistant rate the level of excitement and anxiety of the child in anticipation of the MRI scans. This is done using a Visual Analogue Scale where the rater indicates on two questions how excited the child feels and how tensed the child feels. These measurements are used as a proxy of scanner-related distress. If any of the three raters estimate high scanner-related distress, the MRI visit may be canceled. This procedure is repeated just before commencing the MRI session. Furthermore, the MRI session can be canceled at any time if the child or the parent/guardian indicates that the child does not feel comfortable continuing.

#### 2.2.2 Acquisition

Scans are acquired on a Philips Ingenia 3.0 T CX scanner with a 60 cm bore (Philips Medical Systems, Best, The Netherlands), using a 32-channel SENSE head-coil and operated using software version R530. First, a structural T1-weighted 3D gradient echo scan is acquired, followed by a diffusion-weighted multi-shell multi-band echo planar (EPI) acquisition including two short DWI scans with a reversed phase encoding readout to correct for susceptibility artefacts. Next, multi-band EPI acquisitions are acquired during resting-state, the inhibition task and the emotion task. During the acquisition, the structural T1-weighted scan is visually checked for motion artefacts. If the MR operator regards the scan as unusable due to severe motion artefact, the scan is repeated after emphasizing the instructions to lie still. Prior to the fMRI scans, a short EPI acquisition scan of one dynamic is acquired. MR operators use this scan to visually check the reconstruction for (shimming) artefacts or for placement of the head outside of the field of view. If rescanning is needed, this can come at the expense of the last acquisition as we always ensure that the ethically approved maximal time in the MR scanner is not exceeded.

**Fig. 1:**
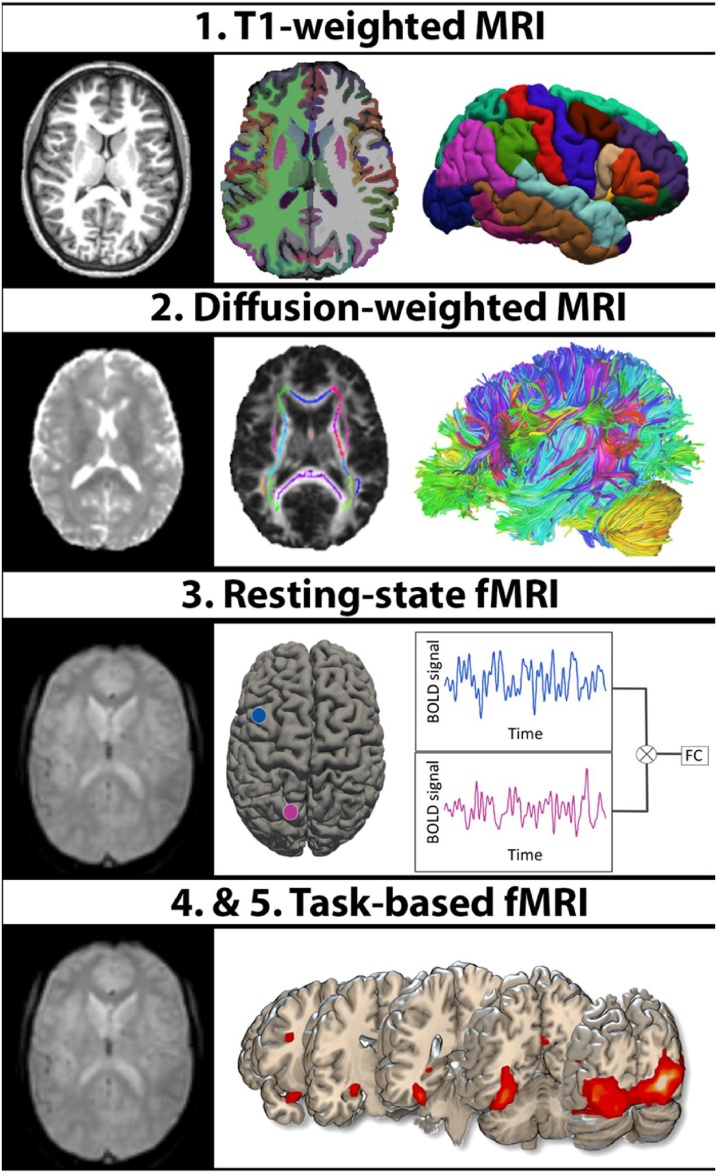
Scan types collected in YOUth in order of acquisition. 1) Original T1-weighted scan (left), with subcortical and cortical brain tissue segmentation (middle) and the cortical regions of interest (right). 2) Diffusion unweighted volume after preprocessing (left); the intersection of the white matter regions (colored) and the skeleton plotted on the FA map (middle); the reconstructed fiber tracts used to create the connectivity maps (right). 3) One dynamic volume of the fMRI scan (left) and a schematic representation of how functional connectivity is computed (right). 4) One dynamic volume of the fMRI scan (left) and task-related activity during the face-processing in the emotion task (right).

The main acquisition parameters are listed in Table 1. See Supplement A.1, for the complete set of acquisition parameters. An illustration of the scan types collected in YOUth can be found in Fig. 1.

**Table 1:** Acquisition parameters YOUth MRI protocol.

Parameters	Structural T1-weighted	DWI	EPI		
			resting-state	inhibition task	emotion task
Acquisition time (m:s)	10:02	8:05	8:07	9:22	6:40
Scan orientation	sagittal	transversal	transversal	transversal	transversal
TR (ms)	10	3500	1000	1000	1000
TE (ms)	4.6	99	25	25	25
Flip angle (degrees)	8	90	65	65	65
Number of slices	*	66	51	51	51
Slice thickness (mm)	*	2.0	2.5	2.5	2.5
Field of view (mm)	240 × 240 × 200	224 × 224	220 × 220	220 × 220	220 × 220
Acquisition matrix	304 × 304	112 × 112	88 × 88	88 × 88	88 × 88
Reconstructed voxel size (mm^3^)	0.75 × 0.75 × 0.80	2.0 × 2.0 × 2.0	2.5 × 2.5 × 2.5	2.5 × 2.5 × 2.5	2.5 × 2.5 × 2.5
Multiband acceleration factor	Off	3	3	3	3
Parallel imaging factor	1.70 (AP)	1.30 (AP)	1.80 (AP)	1.80 (AP)	1.80 (AP)
	1.40 (RL)				
Diffusion directions		105			
b-values (s/mm^2^) [directions]		500 [15]			
		1000 [30]			
		2000 [60]			
		*every 10^th^ scan is a B0-scan*			

Abbreviations: m = minutes; s = seconds; TR = repetition time; TE = echo time; ms = milliseconds; mm = millimeter; AP = anterior-posterior axis; RL = right-left axis; *3D acquisition.

#### 2.2.3 Stimulus presentation

During the scan session, stimuli for fMRI acquisitions are presented using an MRI-compatible 23-inch LCD screen with a resolution of 1080 by 1920 pixels (BOLDscreen, Cambridge Research Systems). During the rs-fMRI acquisition, lights inside the scanner room are turned off and participants are instructed to look at a white fixation cross on a grey screen.

#### 2.2.4 Inhibition task

The stop-signal anticipation task for functional MRI (Zandbelt and Vink, 2010) aims to measure performance and brain activation during actual stopping as well as during the anticipation of stopping. Subjects are presented with three parallel horizontal lines. On each trial, a bar moves at a constant speed from the lower line towards the upper line, reaching the middle line in 800 milliseconds. The main task is to stop the bar as close to the middle line as possible, by pressing a button with the right thumb (i.e. Go trial). Stop trials are identical to Go trials, except that the bar stops moving automatically before reaching the middle line, indicating that a response has to be suppressed (i.e. stop-signal). The probability that such a stop-signal will appear is manipulated across trials and can be anticipated based on three different cues; '0' indicating 0% chance, '*' 22 percent and '**' 33 percent chance the bar will stop on its own. Task difficulty is adjusted to performance in a stepwise fashion, with a varying delay between the stop-signal and the target (i.e. the stop-line) depending on the success of the previous trial, thereby keeping the number of failed and successful trials comparable between subjects and sessions.

#### 2.2.5 Emotion task

The emotion task is aimed at activating face processing areas in the brain. Participants are required to passively view pictures of faces (happy, fearful, or neutral expression) and pictures of houses. The pictures are presented in a pseudorandom order with blocks of face images interspersed with blocks of house images. The stimuli are taken from the Radboud Faces Database (Langner et al., 2010). Stimuli are presented in blocks of 18 s, with four blocks for each of the four stimulus types. Rest periods are modeled as implicit baseline. Because of the short duration of the task, this block-design combined with passive viewing was chosen to ensure a strong contrast between conditions, without noise from behavioral responses. Behavioral data on emotion recognition in the children is measured in another part of YOUth (outside the scanner) during a computer task. To ensure that participants stay awake, they are instructed to press a button in between the block in response to a cue (red circle).

For more information on both fMRI tasks and their usage in the YOUth cohort study, see:

www.uu.nl/en/research/youth-cohort-study.

### 2.3 The YOUth MRI protocol - quality control

In the YOUth study, all children are scanned on the same scanner, with the acquisition parameters kept as stable as possible over the course of the study. Scanner soft- and firmware are only updated when it concerns essential updates with minimal impact on the acquisition. Scanner performance is monitored systematically throughout the YOUth study.

#### 2.3.1 Monitoring scanner performance using human data

Collected MRI-scans of the children are processed immediately after data collection for quality control purposes, on a local server with scripted pipelines. Functional MRI scans are processed using SPM12 (http://www.fil.ion.ucl.ac.uk/spm/). The structural T1-weighted scans are processed using the CAT toolbox (http://www.neuro.uni-jena.de/cat/). DWI scans are processed using the SQUAD-tool running on FSL (Andersson and Sotiropoulos, 2016; Bastiani et al., 2019). Quality control (QC) measures are generated automatically after each scanning session and results are accessible through a web-portal on the local intranet for in-house viewing purposes. These reports consist of different slices generated from the T1-weighted scans to allow for a visual check, with additional statistics like noise- and inhomogeneity-contrast ratios from the CAT toolbox. A single researcher, experienced in quality control, visually checks these reports and this results in a list of scans that are deemed unusable due to inhomogeneity and movement artefacts. In the future, we will also perform a QC on the outer surface reconstruction of the FreeSurfer output to have more information about which scans are unusable. We do not plan to provide quality information at the ROI-level as there is no golden standard for this type of QC yet and depending on the research question different processing software or parcellation atlases can be used. For DWI scans the reports are generated using QUAD (part of FSL’s EDDY QC) and include information on the amount of spatial distortion and artefacts in the scans (Bastiani et al., 2019). For fMRI-scans statistics on movement and signal-to-noise ratio (SNR) are generated, including signal maps for visual inspection. Reports are checked manually after each scanning session and a qualitative assessment is saved as meta-data to the local XNAT storage server (Marcus et al., 2007) together with the raw data. An example of a QC report, generated for each participant, is added in Supplement B.

#### 2.3.2 Monitoring scanner performance using phantom data

Every other week a proton (demi water) spherical phantom (Philips sphere A fluid, doped with CuSO_4_ 1 mL + SH_2_O 60 mg; acetate 2.5 mL; ethanol 5.0 mL; H_3_PO_4_ 4.4 mL; total contents 524 mL) fixed in a standard placeholder is used to acquire a series of scans. These scans include a B0 map to determine the uniformity of the main magnetic field based on two gradient echo images with varying echo time; a B1 map to determine the uniformity of the excitation field based on two gradient echo images with varying repetition time; a 3D gradient echo scan with, and without, the use of gradients and RF excitation; and a dynamic fast field EPI scan (2000 dynamics and 30 dummy scans). After each measurement, data is processed automatically. The output is accessible through a local server and results are inspected to monitor changes over time as well as temporary changes.

#### 2.3.3 Example of data on scanner stability in YOUth

**Fig. 2:**
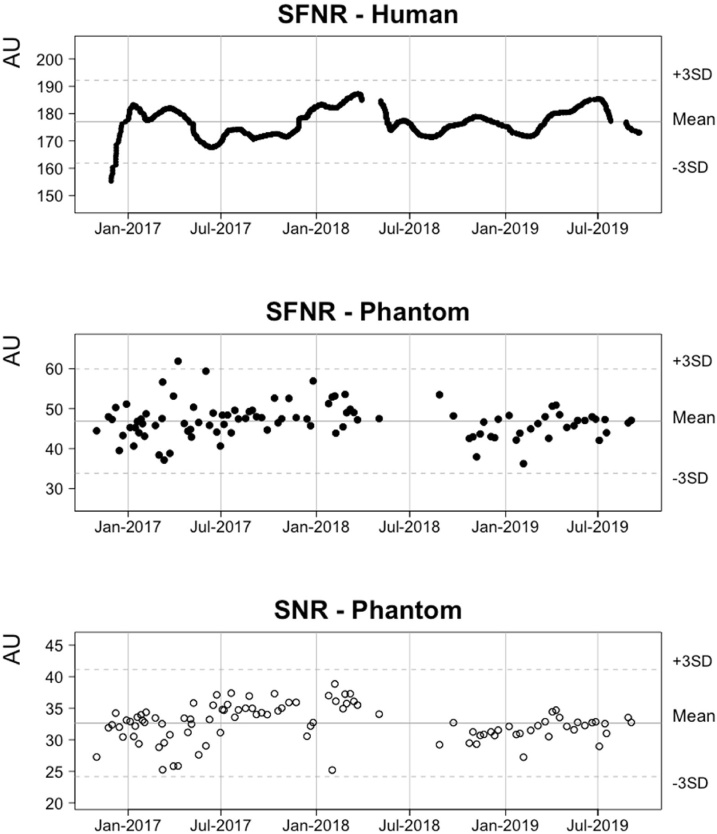
Monitoring scanner performance with human and phantom data using dynamic EPI scans. Data on scanner stability over the course of the study. The solid horizontal line indicates the mean of the signal and the dotted line indicates a threshold of ±3 standard deviations from the mean.

Signal-to-fluctuation-noise ratio (SFNR) is an important measure for estimating the presence of unwanted scanner-related variance in fMRI data (Bennett and Miller, 2010; Murphy et al., 2007) that can e.g. be used as covariate to calibrate multicenter studies (Friedman et al., 2006). A stable scanner would have a high and stable SNR and SFNR. Fig. 2 shows the SFNR calculated from resting-state human data (top row). The human data is derived from the rs-fMRI data collected in the YOUth cohort. The average human data is smoothed by filtering it with a 100-point gaussian window. Fig. 2 also shows the SFNR (middle row) and the SNR (bottom row) derived from the dynamic fast field EPI scan in the phantom data (Friedman and Glover, 2006; Weisskoff, 1996).

### 2.4 The reliability study – Sample and recruitment of adults

To assess the test-retest reliability of the YOUth MRI protocol, we recruited healthy adult volunteers under the premise of MRI protocol development approved by the Medical Ethical Committee. All participants gave written informed consent prior to participation. Test-retest data was collected in adults, in the absence of ethical approval to include YOUth participants for this purpose. Participants were scanned twice with the MRI protocol used in the YOUth children’s cohort study described above. The scan-rescan interval was between 6 and 8 days. The test-retest sample consisted of 17 volunteers (7 male and 10 female) with a mean age of 23 years old (range: 19–31 years old). The participants, most of which were university students, were not given any restrictions regarding food or drink intake.

### 2.5 The reliability study - MRI processing

All scans were visually checked before starting the analyses. If a scan was excluded from analysis, both test and retest scans of the subject were excluded. Only those scans were excluded that had such obvious artefacts or anatomical anomalies that they would have been removed in regular practice. This resulted in sample sizes of 15 or 16 subjects depending on the type of scan. For the reliability-analyses of the T1-weighted scans, one male was excluded due to a structural anomaly. For the analyses of the DWI scans, one female was excluded due to motion artefacts and one female due to extensive spatial distortions. For the analysis of the resting-state MRI data, one female was excluded due to motion artefacts and one male due to an anatomical anomaly. For the task-based fMRI analyses, one male was excluded due to a local artefact and one female was excluded due to missing data.

#### 2.5.1 Processing of structural T1-weighted scans

The T1-weighted test-retest scans were processed using FreeSurfer version 6.0 (freesurfer.net) for automatic brain segmentation and parcellation (Fischl et al., 2002). Global and regional brain measures of subcortical volume, cortical volume, cortical thickness and cortical surface area were extracted. The ROIs established according to the Desikan-Killiany atlas were used for further analysis (Desikan et al., 2006). Besides atlas-based measures of cortical thickness, vertex-wise cortical thickness measures were extracted to include a measure that is independent of a parcellation atlas. For the vertex-wise analysis, cortical thickness of each scan was resampled to an average brain created with FreeSurfer by averaging the first scan of each participant in the test-retest dataset. After resampling, the cortical surface was smoothed with a 3D Gaussian kernel (FWHM =10 mm).

#### 2.5.2 Processing of DWI scans

FSL (version 6.01) in combination with MRtrix (version 3.0) was used to preprocess the DWI scans as described in detail here: B.A.T.M.A.N.: https://osf.io/fkyht/). Preprocessing included gradient direction correction (Leemans and Jones, 2009), eddy current (Andersson and Sotiropoulos, 2016) and susceptibility corrections (Andersson et al., 2003) as well as a correction for Gibbs ringing (Perrone et al., 2015). No correction for signal drift was needed because dynamic stabilization was applied in the acquisition. The results were visually checked and a QC check was performed (using squad, part of FSL). Tract-Based Spatial Statistics (TBSS) were used with the default settings to create a skeletonized version of the fractional anisotropy (FA) and mean diffusivity (MD) values computed from the single tensors (computed using FSL’s DTIFit) that were fitted to the preprocessed multi-shell diffusion data. Global FA and MD values were computed for all skeleton voxels. In addition, average FA and MD values were computed over skeleton voxels from 48 regions of interest (ROIs) selected from the ICBM-DTI-81 white matter (WM) labels atlas (Mori and van Zijl, 2007) similar to (Svatkova et al., 2015).

Connectivity maps were constructed using MRtrix to perform test-retest analysis of the structural network analysis. Here the gray matter (GM) ROIs of the Desikan-Killiany atlas from the FreeSurfer output (generated while processing the T1-weighted scans) were used to define the nodes of the network. Fiber orientation distributions were estimated by deconvolution of the diffusion signal using 8th order spherical harmonics. The response function was obtained using the multi-shell-multi-tissue constrained spherical deconvolution algorithm. For each dataset 5,000,000 streamlines were generated within a seeding area covering the whole brain using deterministic tracking and a FOD-amplitude threshold of 0.05. The number of streamlines was then filtered down to 1,000,000 so that streamline densities better matched the fiber orientation distributions. Connectivity maps were generated by assigning streamlines to the closest node (ROI) found within a 2 mm radius of the streamlines’ endpoints. Streamlines were stored only if they connected two different nodes. Connectivity maps were created based on the number of streamlines and their mean FA for each edge (connection between nodes). Only edges with at least four streamlines in 60 % of the subjects were included in the analysis (de Reus and van den Heuvel, 2013). For these connectivity maps characteristic path length, global efficiency, mean local efficiency and mean strength were calculated (Dimitriadis et al., 2017).

#### 2.5.3 Processing of rs-fMRI scans

Processing of rs-fMRI scans was performed using the CONN toolbox version 18a (Whitfield-Gabrieli and Nieto-Castanon, 2012) and SPM12 (http://www.fil.ion.ucl.ac.uk/spm/) in MATLAB 2015b (The MathWorks Inc., Massachusetts, United States). The structural T1-weighted MRI scans were segmented into cerebrospinal fluid (CSF), GM and WM tissue maps, and registered to MNI-152 space using unified segmentation. The WM and CSF tissue maps were threshold at >50 % and binarized to create tissue masks. The WM masks were eroded by two voxels to reduce the number of voxels at the white-gray matter tissue interface. The CSF tissue masks were constrained to contain only voxels inside the lateral ventricles. Motion correction was performed by realigning the volumes of the rs-fMRI scans to the mean functional volume using a rigid-body transformation in a two-stage approach. The transformation parameters were used to compute frame-wise displacement as an approximation of in-scanner head motion (Power et al., 2012). No slice-timing correction was performed to avoid temporal interpolation of the BOLD signal. Slice-timing correction provides little benefit with fast/short TR or multiband EPI sequences such as used in the current study (TR =1 s, multiband factor = 3), and has no effect on the reliability of functional connectivity estimates (Parker et al., 2016, 2019). The realigned rs-fMRI scans were co-registered with the structural scans using a rigid-body transformation. The structural scans, tissue maps, and rs-fMRI scans were transformed into MNI-152 space and resampled to a 2.0 mm isotropic resolution in a single concatenated transformation step to minimize data-loss as a result of resampling. No spatial smoothing was applied.

Correction for confounding effects was performed using linear regression of the top ten principal components from the BOLD signal of WM and (ventricular) CSF maps (Behzadi et al., 2007; Chai et al., 2012), 24 head motion parameters (Friston et al., 1996; Yan et al., 2013), and scrubbing of a subject-dependent number of frames (Power et al., 2012). Scrubbing of frames with high motion (FD > 0.30 mm) or unusually large whole-brain BOLD signal changes (DVARS Z-score > 3.0) was performed by including a regressor for each of the flagged frames, the preceding frame, and the two following frames (Power et al., 2012). Linear regression was performed on the individual voxels of the brain after quadratic detrending of the BOLD time series to reduce the effects of scanner drift, followed by temporal bandpass filtering at the frequency range of 0.008 to 0.080 Hz (Waheed et al., 2016). All resting-state functional MRI scans were processed independently from each other.

#### 2.5.4 Processing of task-based fMRI scans

Functional MRI scans were processed using SPM12 (http://www.fil.ion.ucl.ac.uk/spm/) in MATLAB 2015b (The MathWorks Inc., Massachusetts, United States). Preprocessing involved realignment, slice timing correction, spatial normalization to MNI-152 space, and smoothing (8 mm full width at half maximum) to correct for inter‐individual differences. Functional images were then submitted to a general linear model.

For both tasks two contrasts were created. For the inhibition task these were: 1) successful stops versus go trials with a stop-signal probability of zero percent, 2) successful stops versus go trials with a stop-signal probability of 20 and 33 percent (from here on referred to as >0% stop-signal probability). For the face processing task, we also created two contrasts: 1) images of faces versus rest, 2) images of faces versus images of houses. Six realignment parameters were added as regressors of no interest to correct for head motion. All data were high‐pass filtered with a cut‐off of 128 s to control for low‐frequency drifts. These analyses produced four (two contrasts per task) t-maps for each participant.

### 2.6 The reliability study – Statistical analysis

Test-retest reliability was quantified with ICCs and their 95 % confidence intervals calculated with the irr package version 0.84.1 in R (https://www.r-project.org/). ICCs were computed using a single measure, absolute-agreement, 2-way random-effects model. Average ICCs were always computed after Fisher’s Z transformation of the individual correlations. Percentage difference (PD) was calculated for each individual and the subsequent mean was calculated from the absolute values of the individual PDs.

#### 2.6.1 Reliability of structural T1-weighted MRI

Global brain measures of cortical and cerebellar volume, cortical thickness and cortical surface area were used to compute mean absolute PDs and ICCs. Next, ICCs were calculated on atlas-based brain measures of subcortical volume, cortical volume, cortical surface area and cortical thickness. Additionally, ICCs were calculated for vertex-wise cortical thickness measures after resampling and smoothing.

#### 2.6.2 Reliability of DWI

For each of the 48 WM ROIs, mean absolute PDs were computed for FA and MD. To determine if there is a relation between certain QC characteristics and reliability of FA and MD, the mean absolute PDs were correlated with SNR (part of the QUAD results), average motion and mean displacement obtained from the QC data. For network analysis, ICCs for FA and the number of streamlines were calculated for each included edge. In addition, ICCs were calculated for the mean characteristic path length, global efficiency, mean local efficiency and mean strength (Dimitriadis et al., 2017).

#### 2.6.3 Reliability of resting-state fMRI

The spatially-averaged BOLD signal was obtained from the unsmoothed and denoised time series for components of major resting-state networks defined in the networks atlas provided by the CONN toolbox (Whitfield-Gabrieli and Nieto-Castanon, 2012; https://web.conn-toolbox.org/; Supplement C, Fig. S1). Functional connectivity estimates were computed using full Pearson correlation between the BOLD signal of two regions. Fisher r-to-Z transformation of the functional connectivity estimates was performed prior to statistical analysis. Test-retest reliability of the Z-transformed functional connectivity estimates was assessed using the ICC as described before. For mean functional connectivity within and between resting-state networks, the ICCs were computed for the averaged Z-transformed functional connectivity estimates across all connections within or between the resting-state network(s).

#### 2.6.4 Reliability of task-based fMRI

##### 2.6.4.1 Behavioral reliability

For the stop-signal task behavioral ICCs were calculated for response times and accuracy. During the emotion task no behavioral data was collected.

##### 2.6.4.2 Imaging reliability

ICCs were computed for each voxel of the brain using the unthresholded t-maps resulting from the statistical analysis in the processing phase. This voxel-wise analysis yielded a 3D matrix of Fisher transformed ICC values. An ROI-analysis was subsequently conducted using the automated anatomical labelling (AAL) template (Tzourio-Mazoyer et al., 2002), generating mean activation levels per AAL region. As these tasks were designed to elicit activation in specific regions of the brain, statistics for selected regions are reported. For the inhibition task, these are bilateral ROIs based on previous research (Vink et al., 2014; Zandbelt et al., 2013), spanning the putamen, motor cortex, and frontal and parietal lobe. As the face/house task is aimed at activating face processing areas in the brain, we report the reliability of occipital, parietal and temporal regions of interest (Passarotti et al., 2003). In addition to statistics for specific ROIs, the mean of ICC values for all voxels across the whole brain are also reported per contrast.

### 2.7 The reliability study - post-hoc analysis: sample size estimations

To better understand the implications of our results for future studies, we did a post-hoc analysis, modelling samples size as a function of effect size Cohen’s D. Power was set at 80 % (beta = 0.2) and the alpha level was set at 0.05. We assumed normally distributed brain measures. Cohen’s D was varied between 0 and 0.5. For each scan type we used the main ICC findings as estimates of reliability, and computed sample size as (z_(1-_***_alpha_***_/2)_ + z_(1-_***_beta_***_)_)^2^/(ICC*Cohen’s D)^2^.

## 3 Results

### 3.1 Reliability of structural T1-weighted MRI

The test-retest reliability of global structural brain measures was high (Table 2). Especially cortical and cerebellar GM volume, intracranial volume and total cortical surface area were highly replicable as indicated by a comparable mean and standard deviation between the two scan sessions, a small mean absolute PD (< 1.43 %) and an excellent ICC (> 0.98). Global measures of cerebellar WM were highly reliable (mean absolute PD < 3.35 %; ICC > 0.90). Average cortical thickness could be reliably measured as well (mean absolute PD < 1.25 %; ICC > 0.74)

**Table 2:** Test-retest statistics of global brain measures.

Global brain measure (mm, mm^2^ or mm^3^)	Mean (SD) *Test*	Mean (SD) *Retest*	Mean absolute PD	ICC [95 % CI]
	***ml***	***ml***	***%***	
Intracranial volume	1484 (258)	1494 (262)	1.11 (1.82)	0.99 [0.98–1.00]
Brain volume without ventricles	1159 (124)	1158 (126)	0.67 (0.31)	1.00 [0.99–1.00]
Left cortical GM	250.6 (21.4)	249.9 (22.6)	1.09 (1.08)	0.98 [0.96–0.99]
Right cortical GM	252.6 (22.3)	251.9 (22.9)	1.43 (1.37)	0.98 [0.93–0.99]
Left cortical WM	227.4 (34.2)	227.8 (35.1)	0.73 (0.69)	1.00 [0.99–1.00]
Right cortical WM	228.4 (35.5)	228.8 (36.2)	0.79 (0.72)	1.00 [0.99–1.00]
Left cerebellum GM	55.82 (5.28)	55.82 (5.20)	0.94 (0.72)	0.99 [0.98–1.00]
Right cerebellum GM	54.79 (5.44)	54.78 (5.44)	0.72 (0.55)	1.00 [0.99–1.00]
Left cerebellum WM	15.25 (1.48)	15.15 (1.66)	3.28 (3.20)	0.90 [0.74–0.96]
Right cerebellum WM	14.48 (1.58)	14.42 (1.85)	3.35 (3.12)	0.93 [0.80–0.97]
	***cm2***	***cm2***	***%***	
Left total surface area	894.2 (95.3)	893.6 (95.6)	0.45 (0.43)	1.00 [0.99–1.00]
Right total surface area	895.3 (96.5)	894.9 (97.1)	0.42 (0.27)	1.00 [1.00–1.00]
	***mm***	***mm***	***%***	
Left average thickness	2.493 (0.056)	2.487 (0.062)	0.88 (0.75)	0.89 [0.72–0.96]
Right average thickness	2.521 (0.052)	2.514 (0.620)	1.25 (1.10)	0.74 [0.41–0.90]

Abbreviations: ml = milliliter; cm = centimeter; mm = millimeter; SD = standard deviation; PD = percentage difference; ICC = intraclass correlation; CI = confidence interval; GM = gray matter; WM = white matter.

**Fig. 3:**
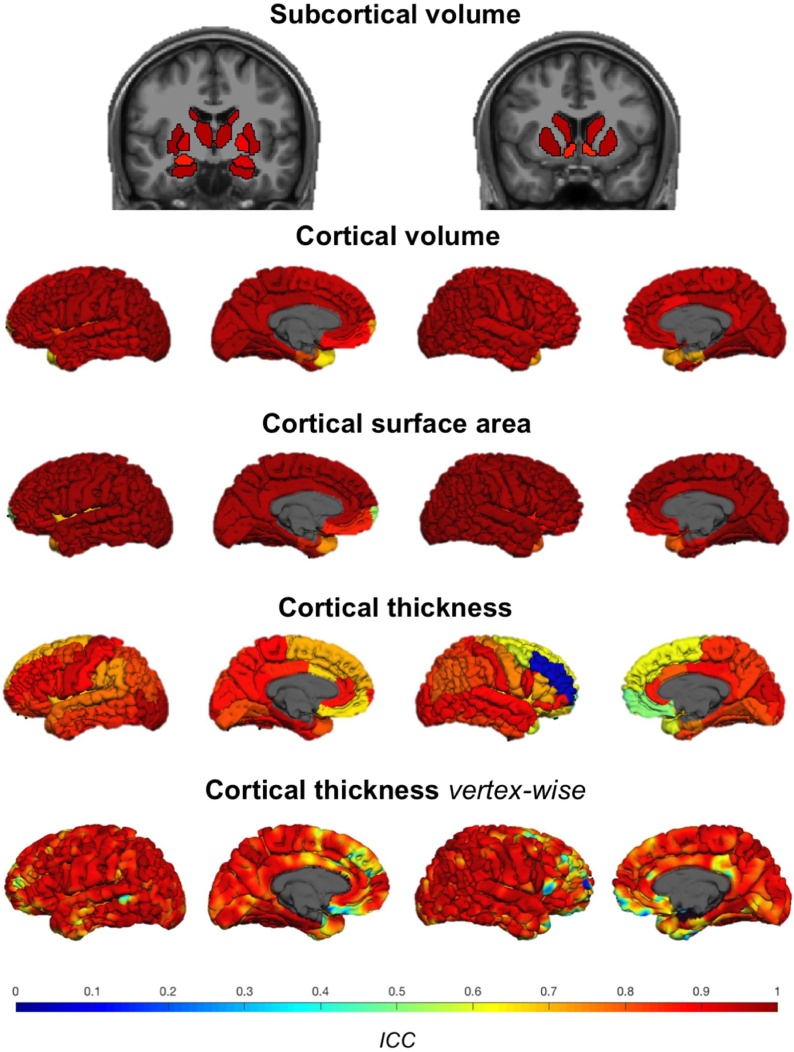
Test-retest ICCs of subcortical and cortical brain measures. The first row shows the ICCs of subcortical volumes on two coronal slices. The slice on the left cuts through the caudate nucleus, thalamus, putamen, pallidum, amygdala and hippocampus. The slice on the right cuts more anterior through the caudate nucleus, putamen and nucleus accumbens. The second, third and fourth row show ICCs of cortical volume, cortical surface area and cortical thickness respectively. The last row shows vertex-wise cortical thickness ICCs. The ICCs of cortical measures are shown on the surface from an outer and medial view with the left hemisphere on the left and the right hemisphere on the right. To visualize the regional test-retest reliability, a model brain was created using the first scan of each participant (Peper et al., 2009; supporting information) and segmented and parcellated with FreeSurfer. For each region or vertex, the ICC was recoded to an RGB color-code using colormap jet in MATLAB.

Fig. 3 shows regional test-retest ICCs for subcortical and cortical brain measures. The ICCs for each region are also listed in Supplement C, Table S1. Regional test-retest ICCs of subcortical volumes were high with an average of 0.95 (ICCs ranging from 0.84 to 0.99) over all regions in both hemispheres. Regional test-retest ICCs for cortical volumes were high with an average of 0.96 (ICCs ranging from 0.65 to 1). Regional test-retest ICCs for cortical surface area were high with an average of 0.98 (ICCs ranging from 0.53 to 1) with the lowest ICC in the left frontal pole. Regional test-retest ICCs for cortical thickness were good with an average of 0.84 (ICCs ranging from 0.07 to 0.97) with the lowest values in the right hemisphere for the rostral middle frontal gyrus (ICC = 0.07), frontal pole (ICC = 0.48) and medial orbitofrontal gyrus (ICC = 0.51). Vertex-wise cortical thickness ICCs were high with an average ICC over all vertices of 0.88.

Taking a closer look at the low ICC in the right rostral middle frontal gyrus, we identified three participants with a large change in cortical thickness between the two scan sessions (0.16, −0.10 and −0.25 mm). We did not find artefacts in the raw scan nor segmentation errors. The vertex-wise analysis confirmed lower reliability in this region suggesting a regional effect unrelated to the parcellation atlas. We did not find evidence for an anterior-posterior gradient in vertex-wise reliability and did not find a pattern when looking at scan date or time. Focusing on the participant with the biggest change between sessions (−0.25 mm), recalculating the ICC without this participant increased the ICC in this region to 0.37 suggesting that the low ICC cannot be explained by a single outlier.

### 3.2 Reliability of DWI

#### 3.2.1 FA and MD

The test-retest reliability and 95 % confidence interval of global skeleton FA and MD was 0.94 (ICCs ranging from 0.83 to 0.98) and 0.87 (ICCs ranging from 0.65 to 0.95), respectively. The mean absolute PD for global FA was 0.86 % and for global MD 1.33 %. For the ROI-based test-retest analysis, the mean ICC for FA was 0.84 with values ranging from 0.51 (found in the pontine crossing tract, a part of the middle cerebellar peduncle) to 0.97 (left anterior corona radiata). The mean ICC for MD found in the test-retest analysis was 0.74, ranging from 0.09 (right cerebral peduncle) to 0.95 (fornix - column and body of fornix). See Supplement C, Table S2 for details.

#### 3.2.2 Relation between scan quality and FA/MD

A significant Pearson correlation (0.60, p = 0.02) was found between the PD computed for SNR and the PD computed for global FA. For global MD the association was not significant (−0.35, p = 0.19). For relative motion, a significant negative correlation was found between the PD for relative motion and the PD for global FA (−0.51, p = 0.05) but not for MD (0.15, p = 0.59). No correlation was found between the PD computed for mean voxel displacement and the PD for FA (−0.12, p = 0.67) or MD (−0.33, p = 0.23). See Supplement C, Table S2 for test-retest results of ROIs from the JHU Atlas.

#### 3.2.3 DWI network analysis

**Fig. 4:**
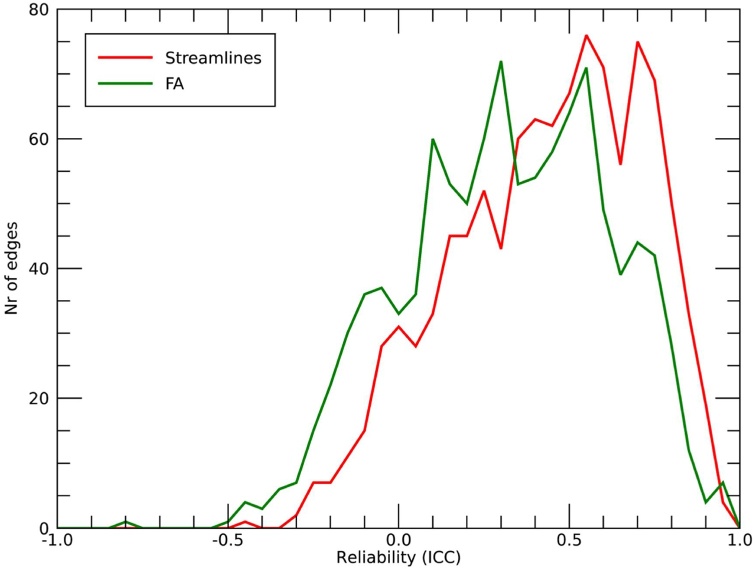
Histogram of the test-retest ICC's of the 1053 included edges. The bin size of the histogram is 0.05.

**Fig. 5:**
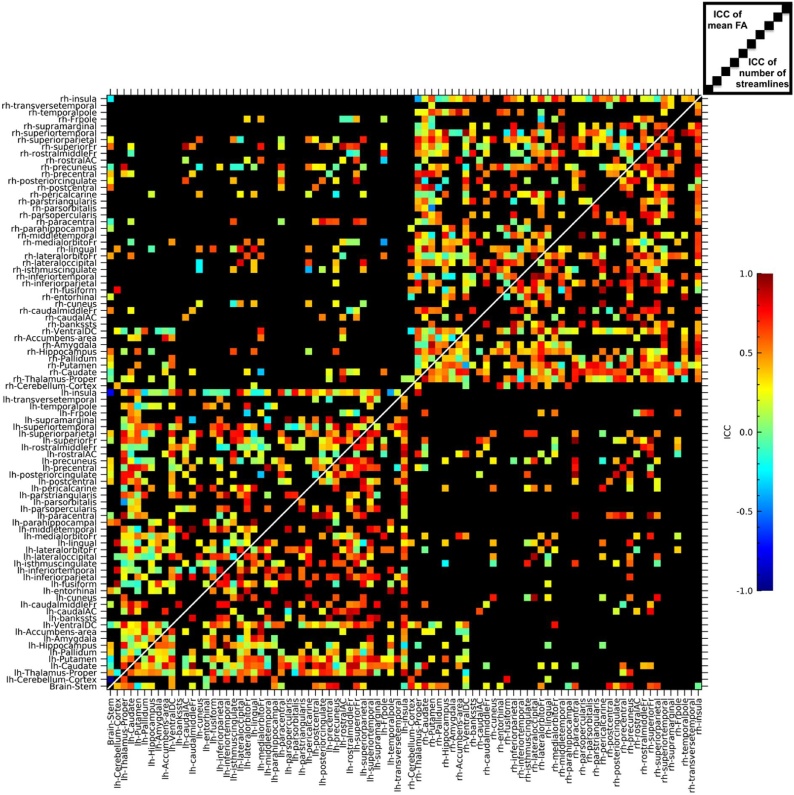
Test-retest ICCs for each individual edge. The upper-left triangle shows the results for the connections weighted with mean FA while the lower-right triangle shows the results for the connections weighted with the number of streamlines. Edges that are colored black were excluded for containing too few streamlines in too many subjects.

The ICCs computed on global network metrics with the connection-weight based on the number of streamlines and for connections weighted using FA are shown in Table 3. A total of 1053 edges were included in the connectivity maps. The mean ICC across edges was 0.52 for the number of streamlines, and 0.39 for the mean FA. Fig. 4 shows the distribution of ICC's of the 1053 edges. Fig. 5 shows the ICCs for the mean FA (upper-left triangle) and for the number of streamlines (lower-right triangle) for each individual edge.

**Table 3:** Test-retest ICCs for global network metrics.

Network metric		Mean (SD)		ICC [95 % CI]
		Test	Retest	
# Streamlines	CPL	1238 (215)	1195 (289)	0.39 [−0.11 to 0.73]
	GE	0.0515 (0.007)	0.0528 (0.009)	0.88 [0.71–0.96]
	MLE	0.0662 (0.009)	0.0686 (0.012)	0.81 [0.56–0.93]
	MS	1.023 (0.130)	1.047 (0.164)	0.91 [0.76–0.97]
FA	CPL	2669 (471)	2745 (527)	0.64 [0.24–0.86]
	GE	0.0679 (0.008)	0.0680 (0.007)	0.58 [0.14–0.83]
	MLE	0.0799 (0.009)	0.0796 (0.009)	0.60 [0.18–0.84]
	MS	1.494 (0.177)	0.1498 (0.163)	0.69 [0.33–0.88]

Abbreviations: CPL = characteristic path length; GE = global efficiency; MLE = mean local efficiency; MS = mean strength; SD = standard deviation; ICC = intraclass correlation; CI = confidence interval; FA = fractional anisotropy.

### 3.3 Reliability of resting-state fMRI

**Fig. 6:**
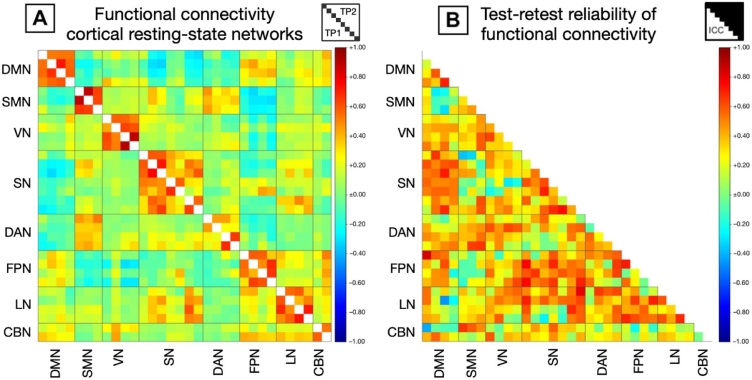
Group-mean functional connectivity (A) and test-retest reliability (B) of functional connectivity for connections between regions of cortical resting-state networks. Abbreviations: DMN = default mode network; SMN = sensorimotor network; VN = visual network; SN = salience network; DAN = dorsal attention network; FPN = frontoparietal network; LN = language network; CBN = cerebellar network; TP1 = estimates from test session; TP2 = estimates from retest session.

Group-mean functional connectivity was highly consistent between scan sessions as indicated by a high correlation between average connectivity at the first and second time point (Pearson’s r = 0.95) with typical higher functional connectivity within resting-state networks and highest functional connectivity between contralateral homotopic regions (Fig. 6A; Supplement C, Table S3). Test-retest reliability of functional connectivity between regions of cortical resting-state networks was moderate (mean ICC = 0.36; ICCs ranging from −0.41 to 0.85; Fig. 6B; Supplement C, Table S4), with moderate to high test-retest reliability of average functional connectivity within cerebral cortical resting-state networks (ICCs ranging from 0.38 to 0.61; Table 4).

**Table 4:** Test-retest reliability of functional connectivity estimates within cortical resting-state networks.

Resting-state network	Mean FC-Z (SD) *Test*	Mean FC-Z (SD) *Retest*	Mean change FC-Z (SD)	ICC [95 % CI]
Default mode	+0.66 (0.23)	+0.67 (0.25)	+0.01 (0.23)	0.61 [0.16–0.85]
Sensorimotor	+1.02 (0.39)	+0.95 (0.37)	–0.06 (0.27)	0.38 [–0.15 to 0.74]
Visual	+0.76 (0.41)	+0.79 (0.38)	+0.03 (0.29)	0.51 [0.02–0.80]
Salience	+0.55 (0.29)	+0.46 (0.30)	–0.09 (0.24)	0.57 [0.10–0.83]
Dorsal attention	+0.39 (0.29)	+0.43 (0.29)	+0.04 (0.25)	0.48 [–0.03 to 0.79]
Frontoparietal	+0.58 (0.26)	+0.65 (0.26)	+0.07 (0.24)	0.41 [–0.11 to 0.76]
Language	+0.70 (0.29)	+0.59 (0.27)	–0.10 (0.23)	0.52 [0.02–0.81]
Cerebellar	+0.65 (0.26)	+0.60 (0.12)	–0.05 (0.28)	–0.01^++^ [–0.50 to 0.49]

Abbreviations: FC-Z = r-to-Z-transformed functional connectivity; SD = standard deviation; ICC = intraclass correlation; CI = confidence interval, **^++^** = lowest ICC.

### 3.4 Reliability of task-based fMRI

#### 3.4.1 Behavioral reliability inhibition task

Only the inhibition task had behavioral measurements in addition to the fMRI data. The ICC for the reaction time, accuracy and response slowing measurements had an average ICC of 0.85 (Table 5). A paired-samples *t*-test was performed on each measure to test for possible learning effects between the two sessions. At the second session, subjects were slower in their incorrect responses, and an increase of the stop probability slope indicates that they slowed down more with increasing stop-signal probability.

**Table 5:** ICC values for behavioral measurements.

Contrast	ICC [95 % CI]	M_1_	M_2_	SD_1_	SD_2_	t	sig
RT correct Go	0.95 [0.85–0.98]	851	856	36	35	−1.91	0.76
RT incorrect Stop	0.92 [0.77–0.97]	829	836	38	34	−2.39	0.03
Stop accuracy	0.71^++^ [0.31–0.90]	0.59	0.59	0.4	0.3	0.77	0.46
Stop signal delay	0.82 [0.53–0.94]	211	209	28	26	0.56	0.58
Stop probability slope	0.91 [0.74–0.97]	91	119	61	64	−2.56	0.02

Abbreviations: ICC = intraclass correlation; CI = confidence interval, **^++^** = lowest ICC.

#### 3.4.2 Imaging reliability inhibition task

Overall ICCs for the first contrast – stop versus go-trials with 0% stop-signal probability – averaged at 0.52. ICCs for the second contrast - stop versus go-trials with >0 % stop-signal probability - were slightly lower, with an average of 0.44. The mean ICC of all voxels across the brain was 0.39 (range −0.76 to 0.92, median 0.47) for the first contrast, 0.37 (range −0.77 to 0.89, median 0.42) for the second. ROI ICCs can be found in Table 6.

**Table 6:** AAL ROI ICC statistics for the inhibition task.

AAL ROI	Stops versus go trials Stop-signal probability = 0	Stops versus go trials Stop-signal probability > 0
	ICC [95 % CI]	ICC [95 % CI]
Precentral gyrus	0.50 [−0.02 to 0.81]	0.49 [−0.03 to 0.80]
Superior frontal gyrus	0.54 [0.04–0.82]	0.48 [−0.04 to 0.80]
Middle frontal gyrus	0.60 [0.13–0.85]	0.48 [−0.04 to 0.80]
Inferior frontal gyrus	0.60 [0.13–0.85]	0.46 [−0.07 to 0.79]
Superior Temporal lobe	0.51 [0.00–0.81]	0.48 [−0.04 to 0.80]
Supplementary motor area	0.55 [0.05–0.83]	0.51 [0.00–0.81]
Paracentral Lobule	0.56 [0.07–0.83]	0.33 [−0.22 to 0.72]
Rolandic Operculum	0.50 [−0.02 to 0.81]	0.47 [−0.06 to 0.79]
Putamen	0.31^++^ [−0.24 to 0.71]	0.23^++^ [−0.32 to 0.66]

Abbreviations: ICC = intraclass correlation; CI = confidence interval, **^++^** = lowest ICC for each contrast.

#### 3.4.3 Imaging reliability face processing task

For the contrast of face versus rest, the average ICC in the selected AAL regions was 0.54. For the contrast of face versus house, the average ICC in the selected AAL regions was 0.64. The mean ICC of all voxels across the brain was 0.34 (range −0.76 to 0.91, median 0.38) for the first contrast, 0.38 (range −0.55 to 0.96, median 0.43) for the second. ROI ICCs can be found in Table 7.

**Table 7:** AAL ROI ICC statistics for faces task.

AAL ROI	Faces versus rest	Faces versus houses
	ICC [95 % CI]	ICC [95 % CI]
Occipital (superior)	0.41^++^ [−0.13 to 0.76]	0.65 [0.21–0.87]
Occipital (middle)	0.53 [0.02–0.82]	0.63 [0.17–0.86]
Occipital (inferior)	0.65 [0.21–0.87]	0.77 [0.42–0.92]
Fusiform gyrus	0.47 [−0.06 to 0.79]	0.68 [0.26–0.88]
Inferior temporal gyrus	0.55 [0.05–0.83]	0.61 [0.14–0.85]
Superior parietal lobe	0.54 [0.04–0.82]	0.65 [0.21–0.87]
Inferior parietal lobe	0.58 [0.10–0.84]	0.43^++^ [−0.11 to 0.77]

Abbreviations: ICC = intraclass correlation; CI = confidence interval, **^++^** = lowest ICC for each contrast.

### 3.5 Post-hoc analysis: sample size estimations

Fig. S2 in Supplement C shows the relationship between the reported ICCs and the sample size needed in future studies to detect an effect of interest with 80 % power and an alpha level of 0.05.

## 4 Discussion

The YOUth MRI protocol was designed to study typical brain development longitudinally in children from 8 years and up. In this paper we provide a detailed description of the MRI acquisition in YOUth and include the test-retest reliability of data collected with this protocol. Global structural brain measures could be estimated with high reliability. Regional structural and functional brain measures in ROIs or specific networks were within the ranges found in literature (outlined below per scan type).

### 4.1 Structural T1-weighted MRI

Regional test-retest ICCs had an average of 0.95 for subcortical volume, 0.96 for cortical volume and 0.98 for cortical surface area. Regional test-retest ICCs for cortical thickness were lower with an average of 0.84 including lower ICCs for some specific regions, mostly in the right hemisphere. Vertex-wise cortical thickness ICCs were, on average, higher with an average ICC over all vertices of 0.88. For most regions, vertex-wise ICCs are comparable to those based on the parcellated region. However, in some regions the vertex-wise ICCs are on average higher than the atlas-based ICC. This difference can be explained by the fact that the between-subject variation for vertex-wise cortical thickness measures is higher than for atlas-based cortical thickness measures in these regions. Our results are in line with other studies that found higher reliability for cortical volume, compared to cortical thickness (Iscan et al., 2015; Liem et al., 2015; Wonderlick et al., 2009). One study also found lower reliability for vertex-wise cortical thickness in the right rostral middle frontal area (Wonderlick et al., 2009). In this study we wanted to have an honest and unbiased estimate of the noise in our brain measures. Therefore, we processed the T1-weighted scans and rescans separately using FreeSurfer’s cross-sectional pipeline. This way, the reliability measures are valid for data obtained from only one measurement too. However, when processing YOUth data, using FreeSurfer’s longitudinal pipeline (Reuter et al., 2012) can improve reliability (Jovicich et al., 2013; Morey et al., 2010).

### 4.2 DWI

Reliable measures of global FA and MD were found. For the ROI-based analysis, the average ICC for FA was 0.84. The average ROI-based ICC for MD was 0.74. Another study also found FA to be more reliable than MD (Duan et al., 2015). At the network level, global network metrics were on average more reliable than metrics at the nodal level, as has been reported before (Dimitriadis et al., 2017). Global network metrics (characteristic path length, global efficiency, mean local efficiency and mean strength) were moderately reliable when weighted by FA, with ICCs between 0.58 and 0.69. The same network metrics were highly reliable when weighted by the number of streamlines, with ICCs between 0.81 and 0.91, with the exception of characteristic path length that was unreliable, ICC = 0.39, comparable to what was found in another study (Cheng et al., 2012). Reliability was lower at the nodal level, with a mean ICC across edges of 0.52 for the number of streamlines, and 0.39 for the mean FA. Numerous methodological choices exist for DWI data, which makes it difficult to directly compare our findings to literature (for an extensive review see: Welton et al., 2015).

### 4.3 Resting-state fMRI

Group-mean functional connectivity was consistent between scan sessions with higher functional connectivity within resting-state networks and highest functional connectivity between contralateral homotopic regions typically observed for cortical resting-state networks. Test-retest reliability of functional connectivity between regions of cortical resting-state networks was moderate with an average ICC over all networks of 0.36, partially due to poor reliability within the cerebellar network. When looking at only cerebral cortical resting-state networks, ICCs were in the range of 0.38 to 0.61. A recent meta-analysis reported an average reliability of 0.29 for functional connectivity on edge-level based on 25 studies (Noble et al., 2019).

### 4.4 Task-based fMRI

The inhibition task had highly reliable behavioral measurements with an average ICC of 0.85. MRI measures during this task had an average ICC over the ROIs of 0.44 and 0.52 for the two task contrasts. MRI measures during the emotion task had an average ICC over the ROIs of 0.54 or 0.64. The contrast between faces and houses generated a more reliable response than the contrast of faces versus rest. These results are in line with ICC values of pre-defined ROIs in other task-based fMRI studies. A meta-analysis of 13 fMRI studies between 2001 and 2009 reported ICCs values in a range from 0.16 to 0.88, with an average reliability of 0.50 (Bennett and Miller, 2010). Similar to our results, reliability generally tends to be best for occipital regions (Koolschijn et al., 2011; Vetter et al., 2015, 2017) and fair to poor for frontal and subcortical regions (Herting et al., 2018). Whole-brain average ICCs were lower than ROI ICCs for both tasks, suggesting that the task contrasts more accurately modulate activity in the targeted ROIs than in other areas. Voxel-wise calculations are a stringent measure of reliability and indicate whether the level of activity in all voxels is consistent between test and retest (Bennett and Miller, 2010).

### 4.5 Factors that determine reliability

In literature, ICCs for functional MRI measures are generally deemed lower compared to structural MRI measures. Our findings are in line with other studies that show that structural MRI brain measures can be measured more reliably than fMRI brain measures. ICC is related to statistical power and therefore the threshold of an acceptable ICC depends on the included sample size and the size of the effect of interest. In MRI research, noise may arise from subject- and MRI-related factors, and their interaction. Effective processing methods can ensure that the effect of noise on the brain measures are kept to a minimum. The impact of methodological choices is reviewed for studies on structural (Mills and Tamnes, 2014; Vijayakumar et al., 2018) and functional brain development (Bennett and Miller, 2010; Herting et al., 2018; Telzer et al., 2018). In-depth investigation of the origin of the noise in our data is beyond the scope of this paper. However, based on the literature we can speculate on possible sources of the noise.

Our acquisition parameters were chosen to create an optimal tradeoff between acquisition duration and SNR/SFNR (e.g. high field strength, isotropic voxels, multiband, scan duration, validated fMRI tasks) and scans were processed using widely-used software. Still, MRI remains a very sensitive measurement technique that inherently has some degree of instability, which may vary per MRI scanner. Consequently, scanner performance is monitored using human and phantom data throughout the YOUth study. Variation is amongst others introduced by scanner drift due to gradient heating and differences between scan sessions with regard to the positioning of participants and variations in shimming (i.e. correcting inhomogeneities of main magnetic field). Therefore, reported results are specific to our scanner, acquisition, processing software and study sample.

Subject movement remains the foremost cause of low reliability of fMRI signals (Gorgolewski et al., 2013b). It has been shown before that residual movement contamination is left in the fMRI BOLD signal even after motion correction (Power et al., 2012). Similarly, our reliability study shows residual variation in DWI scans related to SNR even after correcting for motion. Motion can be a problematic source of variation in longitudinal research as it can be age-related and heritable (Achterberg and van der Meulen, 2019; Savalia et al., 2017; Teeuw et al., 2019; Van Dijk et al., 2012). Therefore, it is important to implement a stringent motion correction technique and QC. Additionally, QC measures, like SNR and SFNR may be included as covariates in DWI and fMRI studies, respectively (Farrell et al., 2007; Friedman and Glover, 2006; Friedman et al., 2006).

For task-based fMRI, additional sources of variation may be introduced by practice effects and compliance to the scanner procedure. Variation induced by the latter can be reduced by familiarizing participants with the MRI environment before the scanning session using a mock scanner as is done within the YOUth cohort. Other subject-related noise can occur due to dehydration (Duning et al., 2005; Kempton et al., 2009; Nakamura et al., 2014; Streitburger et al., 2012), or caffeine intake (Laurienti et al., 2002). Finally, the type and complexity of the task used with an fMRI measurement can greatly affect reliability, with simple motor-movement tasks generally being more reliable than tasks requiring complex cognitive strategies (Gorgolewski et al., 2013a, b).

Scan duration can also greatly affect reliability in fMRI (Birn et al., 2013; Shah et al., 2016; Termenon et al., 2016). A resting-state acquisition duration of approximately 8 min used in the YOUth cohort study is at the minimum recommended duration (Birn et al., 2013). However, the high temporal resolution (TR of 1 s) provides additional sampling points to still achieve a robust measurement within the limited time window. The quality assurance protocol of the YOUth cohort study ensures high temporal SNR (Fig. 1), and might be further improved by early-stage denoising strategies (Adhikari et al., 2018). Denoising strategies to combat the influence of random fluctuations due to physiological noise can result in cleaner estimates of functional connectivity (Caballero-Gaudes and Reynolds, 2017; Parkes et al., 2018), although no optimal strategy currently exists. In some cases, denoising procedures may decrease reliability statistics as reproducible artefacts are also removed (Noble et al., 2019). On a whole, fMRI measurements, such as functional connectivity, are dynamic and state-dependent (Poldrack et al., 2015). As such, longitudinal changes might be due to developmental changes intrinsic to the brain or due to extrinsic factors such as mood, sleep quality, or substance use (Poldrack et al., 2015).

### 4.6 Relevance of reliability results and the relation to power

First, the ICCs reported in this study can be useful to researchers that want to adopt our acquisition parameters (listed in Supplement A). Secondly, it shows how different modalities and processing methods relate to each other in terms of reliability (e.g. FA in ROIs versus FA on edge-level). Lastly, the results can inform researchers that want to apply for data collected in YOUth. Because researchers with all types of research questions can apply for data, in this study we aimed to show reliability measures for each scan using methods that are well-known and widely-used in the field. Our reliability results should not be used to refrain from studying certain brain measures as all of them can be relevant when studying brain development. However, the reliability results can provide guidance when making methodological choices. Accounting for exclusions due to MR safety criteria, scanner-related distress or artefacts, a sample size of 1500 for each type of scan seems sufficient to detect an effect size of 0.2 (Supplement C, Fig. S2). Furthermore, the power analysis shows that it is not advised to apply for small subsamples of the MR data in YOUth, particularly when one is interested in regional measures of DWI on network-level and (rs-)fMRI data.

### 4.7 Limitations

This test-retest study has several limitations. First, the test-retest sample consists of adults, while the YOUth study focuses on development in children. Therefore, the reliability of brain measures found in this study may be considered an overestimation since it does not reflect pediatric data. Consequently, the number of good quality pediatrics scans needed to obtain enough power to detect a certain effect is likely higher than estimated in Fig. S2. In general, more in-scanner head motion is seen in children compared to adults (Thomas et al., 1999; Poldrack et al., 2002; Satterthwaite et al., 2013), but not in all studies (Koolschijn et al., 2011; Alexander -Bloch et al., 2016). Furthermore, processing pediatric data comes with challenges. For example, the processing pipelines used in this study use adult templates as reference for spatial normalization, registration and segmentation. Studies show that using adult templates for pediatric data rather than age-appropriate templates introduces bias in brain measures (Poldrack et al., 2002; Wilke et al., 2002, 2008; Yoon et al., 2009; Fonov et al., 2011). A second limitation can be that the practice effect (for task-fMRI) and compliance effect in this short test-retest period cannot be compared to the three-year scan interval in YOUth. A third limitation is that the test-retest sample size, although in conformance with common practice, is not big enough to mitigate the effect of regional outliers.

### 4.8 Conclusion

It has been shown that neuroimaging studies are often underpowered with the risk of false positive results (Button et al., 2013). Statistical power can be boosted by increasing reliability and sample size. In YOUth, the large sample size together with reasonable to good test-retest reliability increases the probability of finding subtle developmental effects. This paper provides a transparent report of the methodology used in YOUth from MRI acquisition to monitoring quality and reliability. The reliability study shows promising results for the studies that will be done using MRI data collected within the YOUth cohort.

## Declaration of Competing Interest

None.
