# Supplementary material for: The YOUth cohort study: MRI protocol and test-retest reliability in adults
Source: Dev Cogn Neurosci. 2020 Jul 8;45:100816. doi: 10.1016/j.dcn.2020.100816 (PMC7365929; doi:10.1016/j.dcn.2020.100816)
Supplement: Supplementary file 1 [file mmc1.docx]

**The YOUth cohort study: MRI protocol and test-retest reliability in adults**

*Supplement A*

Elizabeth E.L. Buimer*^1^, Pascal Pas*^1^, Rachel M. Brouwer^1^, Martijn Froeling^2^, Hans Hoogduin^2^, Alexander Leemans^2^, Peter Luijten^3^, Bastiaan J. van Nierop^2^, Mathijs Raemaekers^1^, Hugo G. Schnack^1^, Jalmar Teeuw^1^, Matthijs Vink^1,4^, Fredy Visser^5^, Hilleke E. Hulshoff Pol^1^ and René C.W. Mandl^1^

** Elizabeth Buimer and Pascal Pas contributed equally*

^1.^ UMCU Brain Center, University Medical Center Utrecht, University Utrecht, Utrecht, The Netherlands

^2.^ Image Sciences Institute, University Medical Center Utrecht and Utrecht University, Utrecht, the Netherlands.

^3.^ Department of Radiology, University Medical Center Utrecht, Utrecht, The Netherlands

^4.^ Department of Psychology, Utrecht University, Utrecht, The Netherlands

^5.^ Philips Healthcare, Best, The Netherlands

**Corresponding author**

René C. W. Mandl, Utrecht Brain Center, University Medical Center Utrecht, Department of Psychiatry

Heidelberglaan 100 (Room A01.126), 3584CX Utrecht, The Netherlands, +31-0887559705, r.m.mandl@umcutrecht.nl

## **Index**

**A.1 Scan parameters YOUth MRI protocol**

A.1.1 Structural T1-weighted scan

A.1.2 Diffusion-weighted scan anterior-posterior (AP)

A1.3 Diffusion-weighted scan

A1.4 Diffusion-weighted scan posterior-anterior (PA)

A1.5 Functional MRI scan (resting-state followed by 2 task-based scans)

**A.2 Scan parameters YOUth quality control phantom data**

A.2.1 3D FFE SENSE 1 noGRRF (no gradients, no RF)

A.2.2 3D FFE FA1

A.2.3 B0 map

A.2.4 B1 map left

A.2.5 B1 map right

A.2.6 B1 map

A.2.7 Single slice FFE, single shot EPI read-out

## **A.1 Scan parameters YOUth MRI protocol**

### A.1.1 Structural T1-weighted scan

SmartSelect = "yes";

Coil 1 (exclude) = "None";

Uniformity = "CLEAR";

FOV FH (mm) = 240;

AP (mm) = 240;

RL (mm) = 160;

ACQ voxel size FH (mm) = 0.789473712;

AP (mm) = 0.789473712;

RL (mm) = 0.800000012;

Recon voxel size FH (mm) = 0.75;

AP (mm) = 0.75;

RL (mm) = 0.800000012;

Fold-over suppression = "no";

Slice oversampling = "default";

RF select. FOS = "no";

ENCASE enable = "no";

Reconstruction matrix = 320;

SENSE = "yes";

P reduction (AP) = 1.70000005;

S reduction (RL) = 1.39999998;

k-t BLAST = "no";

Stacks = 1;

slices = 200;

slice orientation = "sagittal";

fold-over direction = "AP";

fat shift direction = "F";

Stack Offc. AP (P=+mm) = 17.1326389;

RL (L=+mm) = 3.53828764;

FH (H=+mm) = 33.8094101;

Ang. AP (deg) = 5.58406782;

RL (deg) = 1.79031694;

FH (deg) = 8.69264889;

Free rotatable = "no";

Multi-chunk = "no";

Large table movement = "no";

PlanAlign = "no";

REST slabs = 0;

Shim Size AP (mm) = 104.094604;

RL (mm) = 74.1631546;

FH (mm) = 56.8412132;

Offc. AP (P=+mm) = 22.2333336;

RL (L=+mm) = 2.61283207;

FH (H=+mm) = 59.0336723;

Ang. AP (deg) = 5.45850372;

RL (deg) = -27.9603806;

FH (deg) = 8.913517;

Interactive positioning = "no";

Patient position = "head first";

Patient body position = "head first";

Patient orientation = "supine";

Patient body orientation = "supine";

Scan type = "Imaging";

Scan mode = "3D";

technique = "FFE";

Contrast enhancement = "T1";

Acquisition mode = "cartesian";

Fast Imaging mode = "TFE";

3D non-selective = "no";

shot mode = "multishot";

TFE factor = 179;

3D free factor = "no";

startup echoes = "default";

shot interval = "user defined";

(ms) = 3000;

profile order = "linear";

turbo direction = "Y";

Echoes = 1;

partial echo = "no";

shifted echo = "no";

TE = "shortest";

Flip angle (deg) = 8;

TR = "shortest";

Halfscan = "no";

Water-fat shift = "maximum";

Shim = "PB-volume";

ShimAlign = "no";

mDIXON = "no";

Fat suppression = "no";

Water suppression = "no";

TFE prepulse = "invert";

slice selection = "no";

delay = "shortest";

PSIR = "no";

MTC = "no";

T2prep = "no";

Research prepulse = "no";

Diffusion mode = "no";

Multi-transmit = "no";

SAR mode = "high";

B1 mode = "default";

SAR allow first level = "yes";

PNS mode = "low";

Gradient mode = "default";

SofTone mode = "no";

Cardiac synchronization = "no";

Heart rate > 250 bpm = "no";

Respiratory compensation = "no";

Navigator respiratory comp = "no";

Flow compensation = "no";

fMRI echo stabilisation = "no";

Motion smoothing = "no";

NSA = 1;

Angio / Contrast enh. = "no";

Quantitative flow = "no";

CENTRA = "no";

Manual start = "no";

Dynamic study = "no";

Arterial Spin labeling = "no";

Preparation phases = "auto";

Interactive F0 = "no";

B0 field map = "no";

B1 field map = "no";

MIP/MPR = "no";

SWIp = "no";

Images = "M", (3) "no";

Autoview image = "M";

Calculated images = (4) "no";

Reference tissue = "Grey matter";

Recon compression = "No";

Preset window contrast = "soft";

Reconstruction mode = "immediate";

Save raw data = "no";

Hardcopy protocol = "no";

Image filter = "system default";

Uniformity correction = "no";

Geometry correction = "default";

Elliptical k-space shutter = "default";

IF_info_seperator = 0;

Total scan duration = "10:01.9";

Rel. SNR = 1;

Act. TR/TE (ms) = "10 / 4.7";

ACQ matrix M x P = "304 x 303";

ACQ voxel MPS (mm) = "0.79 / 0.79 / 0.80";

REC voxel MPS (mm) = "0.75 / 0.75 / 0.80";

Scan percentage (%) = 100;

Act. slice gap (mm) = 0;

TFE shots = 200;

TFE dur. shot / acq (ms) = "1889.5 / 1833.1";

Min. TI delay = 963.223999;

Act. WFS (pix) / BW (Hz) = "2.873 / 151.2";

Min. WFS (pix) / Max. BW (Hz) = "0.666 / 652.7";

Local torso SAR = "< 12 %";

Whole body SAR / level = "< 0.2 W/kg / normal";

SED = "< 0.1 kJ/kg";

Max B1+rms = "0.56 uT";

PNS / level = "50 % / normal";

dB/dt = "50.8 T/s";

Sound Pressure Level (dB) = 6.60720539;

### A.1.2 Diffusion-weighted scan anterior-posterior (AP)

SmartSelect = "yes";

Coil 1 (exclude) = "None";

Uniformity = "CLEAR";

FOV RL (mm) = 224;

AP (mm) = 224;

FH (mm) = 132;

ACQ voxel size RL (mm) = 2;

AP (mm) = 2;

Slice thickness (mm) = 2;

Recon voxel size RL (mm) = 2;

AP (mm) = 2;

Fold-over suppression = "no";

Reconstruction matrix = 112;

SENSE = "yes";

P reduction (AP) = 1.29999995;

MB SENSE = "yes";

MB Factor = 3;

k-t BLAST = "no";

Stacks = 1;

type = "parallel";

slices = 66;

slice gap = "user defined";

gap (mm) = 0;

slice orientation = "transverse";

fold-over direction = "AP";

fat shift direction = "P";

Stack Offc. AP (P=+mm) = 15.7327585;

RL (L=+mm) = 2.35860014;

FH (H=+mm) = 24.5950851;

Ang. AP (deg) = 2.43734789;

RL (deg) = -19.9647427;

FH (deg) = 4.51252413;

Free rotatable = "no";

Minimum number of packages = 1;

Slice scan order = "FH";

Large table movement = "no";

PlanAlign = "no";

REST slabs = 0;

Shim Size AP (mm) = 103.028877;

RL (mm) = 76.0707626;

FH (mm) = 56.2589798;

Offc. AP (P=+mm) = 13.9014664;

RL (L=+mm) = 2.41086864;

FH (H=+mm) = 21.8333797;

Ang. AP (deg) = 6.11993647;

RL (deg) = -27.9338512;

FH (deg) = 8.4880743;

Interactive positioning = "no";

Patient position = "head first";

Patient body position = "head first";

Patient orientation = "supine";

Patient body orientation = "supine";

Scan type = "Imaging";

Scan mode = "MS";

technique = "SE";

Modified SE = "no";

Acquisition mode = "cartesian";

Fast Imaging mode = "EPI";

shot mode = "single-shot";

Echoes = 1;

partial echo = "no";

TE = "user defined";

(ms) = 99;

Flip angle (deg) = 90;

TR = "user defined";

(ms) = 3500;

Halfscan = "yes";

factor = 0.655172408;

Water-fat shift = "minimum";

Shim = "PB-volume";

ShimAlign = "no";

mDIXON = "no";

Fat suppression = "SPIR";

strength = "strong";

frequency offset = "default";

Grad Rev Fat suppr = "yes";

Water suppression = "no";

BB pulse = "no";

MTC = "no";

Research prepulse = "no";

Diffusion mode = "DWI";

sequence = "SE";

gradient duration = "maximum";

gradient overplus = "no";

direction = "M", "P", "S";

nr of b-factors = 2;

b-factor order = "ascending";

max b-factor = 1000;

average high b = "user defined";

b-factor averages = "(0) 1",

"(1000) 1", "", "", "", "",

"", "", "", "", "", "", "",

"", "", "", "", "", "", "",

"", "", "", "", "", "", "",

"", "", "", "", "";

Multi-transmit = "no";

SAR mode = "high";

B1 mode = "default";

SAR allow first level = "yes";

PNS mode = "high";

Gradient mode = "default";

SofTone mode = "user defined";

factor = 1.29999995;

Cardiac synchronization = "no";

Heart rate > 250 bpm = "no";

Respiratory compensation = "no";

Navigator respiratory comp = "no";

Flow compensation = "no";

Temporal slice spacing = "default";

NSA = 1;

Manual start = "no";

Dynamic study = "no";

Arterial Spin labeling = "no";

Preparation phases = "auto";

Interactive F0 = "no";

B0 field map = "no";

B1 field map = "no";

MIP/MPR = "no";

Images = "M", (3) "no";

Autoview image = "M";

Calculated images = (4) "no";

Reference tissue = "White matter";

Recon compression = "No";

Preset window contrast = "soft";

Reconstruction mode = "immediate";

Save raw data = "no";

Hardcopy protocol = "no";

Image filter = "system default";

Uniformity correction = "no";

Geometry correction = "default";

IF_info_seperator = 1634755923;

### A1.3 Diffusion-weighted scan

SmartSelect = "yes";

Coil 1 (exclude) = "None";

Uniformity = "CLEAR";

FOV RL (mm) = 224;

AP (mm) = 224;

FH (mm) = 132;

ACQ voxel size RL (mm) = 2;

AP (mm) = 2;

Slice thickness (mm) = 2;

Recon voxel size RL (mm) = 2;

AP (mm) = 2;

Fold-over suppression = "no";

Reconstruction matrix = 112;

SENSE = "yes";

P reduction (AP) = 1.29999995;

MB SENSE = "yes";

MB Factor = 3;

k-t BLAST = "no";

Stacks = 1;

type = "parallel";

slices = 66;

slice gap = "user defined";

gap (mm) = 0;

slice orientation = "transverse";

fold-over direction = "AP";

fat shift direction = "P";

Stack Offc. AP (P=+mm) = 15.7327585;

RL (L=+mm) = 2.35860014;

FH (H=+mm) = 24.5950851;

Ang. AP (deg) = 2.43734789;

RL (deg) = -19.9647427;

FH (deg) = 4.51252413;

Free rotatable = "no";

Minimum number of packages = 1;

Slice scan order = "FH";

Large table movement = "no";

PlanAlign = "no";

REST slabs = 0;

Shim Size AP (mm) = 103.028877;

RL (mm) = 76.0707626;

FH (mm) = 56.2589798;

Offc. AP (P=+mm) = 13.9014664;

RL (L=+mm) = 2.41086864;

FH (H=+mm) = 21.8333797;

Ang. AP (deg) = 6.11993647;

RL (deg) = -27.9338512;

FH (deg) = 8.4880743;

Interactive positioning = "no";

Patient position = "head first";

Patient body position = "head first";

Patient orientation = "supine";

Patient body orientation = "supine";

Scan type = "Imaging";

Scan mode = "MS";

technique = "SE";

Modified SE = "no";

Acquisition mode = "cartesian";

Fast Imaging mode = "EPI";

shot mode = "single-shot";

Echoes = 1;

partial echo = "no";

TE = "user defined";

(ms) = 99;

Flip angle (deg) = 90;

TR = "user defined";

(ms) = 3500;

Halfscan = "yes";

factor = 0.655172408;

Water-fat shift = "minimum";

Shim = "PB-volume";

ShimAlign = "no";

mDIXON = "no";

Fat suppression = "SPIR";

strength = "strong";

frequency offset = "default";

Grad Rev Fat suppr = "yes";

Water suppression = "no";

BB pulse = "no";

MTC = "no";

Research prepulse = "no";

Diffusion mode = "DTI";

sequence = "SE";

gradient duration = "maximum";

gradient overplus = "no";

directional resolution = "from file";

average high b = "user defined";

b-factor averages = "(0) 1",

"(1000) 1", "(2000) 1", "(0) 1",

"(500) 1", "", "", "", "",

"", "", "", "", "", "", "",

"", "", "", "", "", "", "",

"", "", "", "", "", "", "",

"", "";

Multi-transmit = "no";

SAR mode = "high";

B1 mode = "default";

SAR allow first level = "yes";

PNS mode = "high";

Gradient mode = "default";

SofTone mode = "user defined";

factor = 1.29999995;

Cardiac synchronization = "no";

Heart rate > 250 bpm = "no";

Respiratory compensation = "no";

Navigator respiratory comp = "no";

Flow compensation = "no";

Temporal slice spacing = "default";

NSA = 1;

Manual start = "no";

Dynamic study = "no";

dyn stabilization = "regular";

Arterial Spin labeling = "no";

Preparation phases = "auto";

Interactive F0 = "no";

B0 field map = "no";

B1 field map = "no";

MIP/MPR = "no";

Images = "M", (3) "no";

Autoview image = "M";

Calculated images = (4) "no";

Reference tissue = "White matter";

Recon compression = "No";

Preset window contrast = "soft";

Reconstruction mode = "immediate";

Save raw data = "no";

Hardcopy protocol = "no";

Image filter = "system default";

Uniformity correction = "no";

Geometry correction = "default";

IF_info_seperator = 1634755923;

### A1.4 Diffusion-weighted scan posterior-anterior (PA)

SmartSelect = "yes";

Coil 1 (exclude) = "None";

Uniformity = "CLEAR";

FOV RL (mm) = 224;

AP (mm) = 224;

FH (mm) = 132;

ACQ voxel size RL (mm) = 2;

AP (mm) = 2;

Slice thickness (mm) = 2;

Recon voxel size RL (mm) = 2;

AP (mm) = 2;

Fold-over suppression = "no";

Reconstruction matrix = 112;

SENSE = "yes";

P reduction (AP) = 1.29999995;

MB SENSE = "yes";

MB Factor = 3;

k-t BLAST = "no";

Stacks = 1;

type = "parallel";

slices = 66;

slice gap = "user defined";

gap (mm) = 0;

slice orientation = "transverse";

fold-over direction = "AP";

fat shift direction = "A";

Stack Offc. AP (P=+mm) = 15.7327585;

RL (L=+mm) = 2.35860014;

FH (H=+mm) = 24.5950851;

Ang. AP (deg) = 2.43734789;

RL (deg) = -19.9647427;

FH (deg) = 4.51252413;

Free rotatable = "no";

Minimum number of packages = 1;

Slice scan order = "FH";

Large table movement = "no";

PlanAlign = "no";

REST slabs = 0;

Shim Size AP (mm) = 103.028877;

RL (mm) = 76.0707626;

FH (mm) = 56.2589798;

Offc. AP (P=+mm) = 13.9014664;

RL (L=+mm) = 2.41086864;

FH (H=+mm) = 21.8333797;

Ang. AP (deg) = 6.11993647;

RL (deg) = -27.9338512;

FH (deg) = 8.4880743;

Interactive positioning = "no";

Patient position = "head first";

Patient body position = "head first";

Patient orientation = "supine";

Patient body orientation = "supine";

Scan type = "Imaging";

Scan mode = "MS";

technique = "SE";

Modified SE = "no";

Acquisition mode = "cartesian";

Fast Imaging mode = "EPI";

shot mode = "single-shot";

Echoes = 1;

partial echo = "no";

TE = "user defined";

(ms) = 99;

Flip angle (deg) = 90;

TR = "user defined";

(ms) = 3500;

Halfscan = "yes";

factor = 0.655172408;

Water-fat shift = "minimum";

Shim = "PB-volume";

ShimAlign = "no";

mDIXON = "no";

Fat suppression = "SPIR";

strength = "strong";

frequency offset = "default";

Grad Rev Fat suppr = "yes";

Water suppression = "no";

BB pulse = "no";

MTC = "no";

Research prepulse = "no";

Diffusion mode = "DWI";

sequence = "SE";

gradient duration = "maximum";

gradient overplus = "no";

direction = "M", "P", "S";

nr of b-factors = 2;

b-factor order = "ascending";

max b-factor = 1000;

average high b = "user defined";

b-factor averages = "(0) 1",

"(1000) 1", "", "", "", "",

"", "", "", "", "", "", "",

"", "", "", "", "", "", "",

"", "", "", "", "", "", "",

"", "", "", "", "";

Multi-transmit = "no";

SAR mode = "high";

B1 mode = "default";

SAR allow first level = "yes";

PNS mode = "high";

Gradient mode = "default";

SofTone mode = "user defined";

factor = 1.29999995;

Cardiac synchronization = "no";

Heart rate > 250 bpm = "no";

Respiratory compensation = "no";

Navigator respiratory comp = "no";

Flow compensation = "no";

Temporal slice spacing = "default";

NSA = 1;

Manual start = "no";

Dynamic study = "no";

Arterial Spin labeling = "no";

Preparation phases = "auto";

Interactive F0 = "no";

B0 field map = "no";

B1 field map = "no";

MIP/MPR = "no";

Images = "M", (3) "no";

Autoview image = "M";

Calculated images = (4) "no"

Reference tissue = "White matter";

Recon compression = "No";

Preset window contrast = "soft";

Reconstruction mode = "immediate";

Save raw data = "no";

Hardcopy protocol = "no";

Image filter = "system default";

Uniformity correction = "no";

Geometry correction = "default";

IF_info_seperator = 1634755923;

### A1.5 Functional MRI scan (resting-state followed by 2 task-based scans)

SmartSelect = "yes";

Coil 1 (exclude) = "None";

Uniformity = "CLEAR";

FOV RL (mm) = 219.99408;

AP (mm) = 219.99408;

FH (mm) = 127.5;

ACQ voxel size RL (mm) = 2.5;

AP (mm) = 2.5;

Slice thickness (mm) = 2.5;

Recon voxel size RL (mm) = 2.291605;

AP (mm) = 2.291605;

Fold-over suppression = "no";

Reconstruction matrix = 96;

SENSE = "yes";

P reduction (AP) = 1.79999995;

MB SENSE = "yes";

MB Factor = 3;

k-t BLAST = "no";

Stacks = 1;

type = "parallel";

slices = 51;

slice gap = "user defined";

gap (mm) = 0;

slice orientation = "transverse";

fold-over direction = "AP";

fat shift direction = "P";

Stack Offc. AP (P=+mm) = 14.7376213;

RL (L=+mm) = 1.19954967;

FH (H=+mm) = 20.3705826;

Ang. AP (deg) = 0.827678025;

RL (deg) = -22.5936832;

FH (deg) = 0.326388508;

Free rotatable = "no";

Minimum number of packages = 1;

Slice scan order = "FH";

Large table movement = "no";

PlanAlign = "no";

REST slabs = 0;

Shim Size AP (mm) = 96.8558731;

RL (mm) = 82.2925797;

FH (mm) = 58.4264908;

Offc. AP (P=+mm) = 22.2233238;

RL (L=+mm) = -0.23078306;

FH (H=+mm) = 25.6794434;

Ang. AP (deg) = 0.912907779;

RL (deg) = -25.1918774;

FH (deg) = 0.384137094;

Interactive positioning = "no";

Patient position = "head first";

Patient body position = "head first";

Patient orientation = "supine";

Patient body orientation = "supine";

Scan type = "Imaging";

Scan mode = "MS";

technique = "FFE";

Contrast enhancement = "no";

Acquisition mode = "cartesian";

Fast Imaging mode = "EPI";

shot mode = "single-shot";

Echoes = 1;

partial echo = "no";

shifted echo = "no";

TE = "user defined";

(ms) = 25;

Flip angle (deg) = 65;

TR = "user defined";

(ms) = 1000;

Halfscan = "no";

Water-fat shift = "user defined";

(pixels) = 13;

Shim = "PB-volume";

ShimAlign = "no";

mDIXON = "no";

Fat suppression = "SPIR";

strength = "strong";

frequency offset = "default";

Water suppression = "no";

MTC = "no";

Research prepulse = "no";

Diffusion mode = "no";

Multi-transmit = "no";

SAR mode = "high";

B1 mode = "default";

SAR allow first level = "yes";

PNS mode = "moderate";

Gradient mode = "maximum";

SofTone mode = "no";

Cardiac synchronization = "no";

Heart rate > 250 bpm = "no";

Respiratory compensation = "no";

Navigator respiratory comp = "no";

Flow compensation = "no";

Temporal slice spacing = "default";

fMRI echo stabilisation = "no";

NSA = 1;

Angio / Contrast enh. = "no";

Quantitative flow = "no";

Manual start = "no";

Dynamic study = "individual";

dyn scans = 480;

dyn scan times = "shortest";

fov time mode = "default";

dummy scans = 8;

immediate subtraction = "no";

fast next scan = "no";

synch. ext. device = "yes";

start at dyn. = 1;

interval (dyn) = 479;

dyn stabilization = "enhanced";

prospect. motion corr. = "no";

Keyhole = "no";

Arterial Spin labeling = "no";

Preparation phases = "auto";

Interactive F0 = "no";

B0 field map = "no";

B1 field map = "no";

MIP/MPR = "no";

SWIp = "no";

Images = "M", (3) "no";

Autoview image = "M";

Calculated images = (4) "no";

Reference tissue = "Grey matter";

Recon compression = "No";

Preset window contrast = "soft";

Reconstruction mode = "real time";

reuse memory = "no";

Save raw data = "no";

Hardcopy protocol = "no";

Image filter = "system default";

Uniformity correction = "no";

Geometry correction = "none";

IF_info_seperator = 1634755923;

## **A.2 Scan parameters YOUth quality control phantom data**

### A.2.1 3D FFE SENSE 1 noGRRF (no gradients, no RF)

SmartSelect = "yes";

Coil 1 (exclude) = "None";

Uniformity = "CLEAR";

FOV AP (mm) = 150;

RL (mm) = 150;

FH (mm) = 16.5;

ACQ voxel size AP (mm) = 1.5;

RL (mm) = 1.5151515;

FH (mm) = 1.5;

Recon voxel size AP (mm) = 0.9375;

RL (mm) = 0.9375;

FH (mm) = 1.5;

Fold-over suppression = "no";

Slice oversampling = "default";

RF select. FOS = "no";

ENCASE enable = "no";

Reconstruction matrix = 160;

SENSE = "yes";

P reduction (RL) = 1;

S reduction (FH) = 1;

k-t BLAST = "no";

Stacks = 1;

slices = 11;

slice orientation = "transverse";

fold-over direction = "RL";

fat shift direction = "P";

Stack Offc. AP (P=+mm) = 1.76040018;

RL (L=+mm) = -0.822219431;

FH (H=+mm) = -0.297277153;

Ang. AP (deg) = 0;

RL (deg) = -0;

FH (deg) = -0;

Free rotatable = "no";

Multi-chunk = "no";

Large table movement = "no";

PlanAlign = "no";

REST slabs = 0;

Interactive positioning = "no";

Patient position = "head first";

Patient body position = "head first";

Patient orientation = "supine";

Patient body orientation = "supine";

Scan type = "Imaging";

Scan mode = "3D";

technique = "FFE";

loop order = "zy_order";

Contrast enhancement = "T1";

Acquisition mode = "cartesian";

Fast Imaging mode = "none";

3D non-selective = "no";

Echoes = 1;

partial echo = "no";

shifted echo = "no";

TE = "user defined";

(ms) = 3;

Flip angle (deg) = 10;

TR = "user defined";

(ms) = 5.5;

Halfscan = "no";

Water-fat shift = "minimum";

RF Shims = "fixed";

Shim = "manual";

Manual shim reset = "yes";

X shim value = 0;

Y shim value = 0;

Z shim value = 0;

Z2 shim value = 0;

ZX shim value = 0;

ZY shim value = 0;

X2-Y2 shim value = 0;

2XY shim value = 0;

mDIXON = "no";

Fat suppression = "no";

Water suppression = "no";

MTC = "no";

Research prepulse = "no";

Diffusion mode = "no";

Multi-transmit = "yes";

Transmit channels = "both";

SAR mode = "low";

B1 mode = "default";

SAR allow first level = "yes";

Patient pregnancy = "no";

Patient WB SAR [W/kg] = 0;

Patient Head SAR [W/kg] = 0;

Patient max. dB/dt [T/s] = 0;

Max slewrate [T/m/s] = 0;

Max. B1+rms [uT] = 0;

PNS mode = "high";

Gradient mode = "maximum";

SofTone mode = "no";

Cardiac synchronization = "no";

Heart rate > 250 bpm = "no";

Respiratory compensation = "no";

Navigator respiratory comp = "no";

Flow compensation = "no";

fMRI echo stabilisation = "no";

NSA = 1;

Angio / Contrast enh. = "no";

Quantitative flow = "no";

Manual start = "no";

Dynamic study = "individual";

dyn scans = 2;

dyn scan times = "user defined";

(mm:ss) = "shortest (00:00.0)",

"00:20.0", "_", "_", "_",

"_", "_", "_", "_", "_", "_",

"_", "_", "_", "_", "_", "_",

"_", "_", "_", "_", "_", "_",

"_", "_", "_", "_", "_", "_",

"_", "_", "_", "_", "_", "_",

"_", "_", "_", "_", "_", "_",

"_", "_", "_", "_", "_", "_",

"_", "_", "_", "_", "_", "_",

"_", "_", "_", "_", "_", "_",

"_", "_", "_", "_", "_";

fov time mode = "default";

dummy scans = 0;

immediate subtraction = "no";

fast next scan = "no";

synch. ext. device = "no";

dyn stabilization = "no";

prospect. motion corr. = "no";

Keyhole = "no";

Arterial Spin labeling = "no";

Preparation phases = "full";

Interactive F0 = "no";

B0 field map = "no";

B1 field map = "no";

MIP/MPR = "no";

SWIp = "no";

Images = "M", "P", (2) "no";

Autoview image = "M";

Calculated images = (4) "no";

Reference tissue = "Grey matter";

Recon compression = "No";

Preset window contrast = "soft";

Reconstruction mode = "immediate";

Save raw data = "yes";

Hardcopy protocol = "no";

Image filter = "system default";

Uniformity correction = "no";

Geometry correction = "default";

Elliptical k-space shutter = "no";

IF_info_seperator = 0;

Research Options used = "28";

Total scan duration = "00:27.6";

Rel. SNR = 1;

Act. TR/TE (ms) = "5.5 / 3.0";

Dyn. scan time = "00:07.6";

Time to k0 = "00:03.8";

ACQ matrix M x P = "100 x 99";

ACQ voxel MPS (mm) = "1.50 / 1.52 / 1.50";

REC voxel MPS (mm) = "0.94 / 0.94 / 1.50";

Scan percentage (%) = 99;

Act. slice gap (mm) = 0;

Act. WFS (pix) / BW (Hz) = "0.243 / 1785.7";

Min. WFS (pix) / Max. BW (Hz) = "0.222 / 1953.1";

Min. TR/TE (ms) = "4.4 / 1.15";

Head SAR = "< 15 %";

Whole body SAR / level = "0.0 W/kg / normal";

SED = " 0.0 kJ/kg";

Coil Power = "15 %";

Max B1+rms = "0.91 uT";

PNS / level = "67 % / normal";

dB/dt = "68.5 T/s";

Sound Pressure Level (dB) = 16.7623653;

### A.2.2 3D FFE_FA1

SmartSelect = "yes";

Coil 1 (exclude) = "None";

Uniformity = "CLEAR";

FOV AP (mm) = 150;

RL (mm) = 150;

FH (mm) = 16.5;

ACQ voxel size AP (mm) = 1.5;

RL (mm) = 1.5151515;

FH (mm) = 1.5;

Recon voxel size AP (mm) = 0.9375;

RL (mm) = 0.9375;

FH (mm) = 1.5;

Fold-over suppression = "no";

Slice oversampling = "default";

RF select. FOS = "no";

ENCASE enable = "no";

Reconstruction matrix = 160;

SENSE = "yes";

P reduction (RL) = 1;

S reduction (FH) = 1;

k-t BLAST = "no";

Stacks = 1;

slices = 11;

slice orientation = "transverse";

fold-over direction = "RL";

fat shift direction = "P";

Stack Offc. AP (P=+mm) = 1.76040018;

RL (L=+mm) = -0.822219431;

FH (H=+mm) = -0.297277153;

Ang. AP (deg) = 0;

RL (deg) = -0;

FH (deg) = -0;

Free rotatable = "no";

Multi-chunk = "no";

Large table movement = "no";

PlanAlign = "no";

REST slabs = 0;

Interactive positioning = "no";

Patient position = "head first";

Patient body position = "head first";

Patient orientation = "supine";

Patient body orientation = "supine";

Scan type = "Imaging";

Scan mode = "3D";

technique = "FFE";

loop order = "zy_order";

Contrast enhancement = "T1";

Acquisition mode = "cartesian";

Fast Imaging mode = "none";

3D non-selective = "no";

Echoes = 1;

partial echo = "no";

shifted echo = "no";

TE = "user defined";

(ms) = 3;

Flip angle (deg) = 10;

TR = "user defined";

(ms) = 5.5;

Halfscan = "no";

Water-fat shift = "minimum";

RF Shims = "fixed";

Shim = "manual";

Manual shim reset = "yes";

X shim value = 0;

Y shim value = 0;

Z shim value = 0;

Z2 shim value = 0;

ZX shim value = 0;

ZY shim value = 0;

X2-Y2 shim value = 0;

2XY shim value = 0;

mDIXON = "no";

Fat suppression = "no";

Water suppression = "no";

MTC = "no";

Research prepulse = "no";

Diffusion mode = "no";

Multi-transmit = "yes";

Transmit channels = "both";

SAR mode = "low";

B1 mode = "default";

SAR allow first level = "yes";

Patient pregnancy = "no";

Patient WB SAR [W/kg] = 0;

Patient Head SAR [W/kg] = 0;

Patient max. dB/dt [T/s] = 0;

Max slewrate [T/m/s] = 0;

Max. B1+rms [uT] = 0;

PNS mode = "high";

Gradient mode = "maximum";

SofTone mode = "no";

Cardiac synchronization = "no";

Heart rate > 250 bpm = "no";

Respiratory compensation = "no";

Navigator respiratory comp = "no";

Flow compensation = "no";

fMRI echo stabilisation = "no";

NSA = 1;

Angio / Contrast enh. = "no";

Quantitative flow = "no";

Manual start = "no";

Dynamic study = "individual";

dyn scans = 2;

dyn scan times = "user defined";

(mm:ss) = "shortest (00:00.0)",

"00:20.0", "_", "_", "_",

"_", "_", "_", "_", "_", "_",

"_", "_", "_", "_", "_", "_",

"_", "_", "_", "_", "_", "_",

"_", "_", "_", "_", "_", "_",

"_", "_", "_", "_", "_", "_",

"_", "_", "_", "_", "_", "_",

"_", "_", "_", "_", "_", "_",

"_", "_", "_", "_", "_", "_",

"_", "_", "_", "_", "_", "_",

"_", "_", "_", "_", "_";

fov time mode = "default";

dummy scans = 0;

immediate subtraction = "no";

fast next scan = "no";

synch. ext. device = "no";

dyn stabilization = "no";

prospect. motion corr. = "no";

Keyhole = "no";

Arterial Spin labeling = "no";

Preparation phases = "full";

Interactive F0 = "no";

B0 field map = "no";

B1 field map = "no";

MIP/MPR = "no";

SWIp = "no";

Images = "M", "P", (2) "no";

Autoview image = "M";

Calculated images = (4) "no";

Reference tissue = "Grey matter";

Recon compression = "No";

Preset window contrast = "soft";

Reconstruction mode = "immediate";

Save raw data = "yes";

Hardcopy protocol = "no";

Image filter = "system default";

Uniformity correction = "no";

Geometry correction = "default";

Elliptical k-space shutter = "no";

IF_info_seperator = 0;

Research Options used = "28";

Total scan duration = "00:27.6";

Rel. SNR = 1;

Act. TR/TE (ms) = "5.5 / 3.0";

Dyn. scan time = "00:07.6";

Time to k0 = "00:03.8";

ACQ matrix M x P = "100 x 99";

ACQ voxel MPS (mm) = "1.50 / 1.52 / 1.50";

REC voxel MPS (mm) = "0.94 / 0.94 / 1.50";

Scan percentage (%) = 99;

Act. slice gap (mm) = 0;

Act. WFS (pix) / BW (Hz) = "0.243 / 1785.7";

Min. WFS (pix) / Max. BW (Hz) = "0.222 / 1953.1";

Min. TR/TE (ms) = "4.4 / 1.15";

Head SAR = "< 15 %";

Whole body SAR / level = "0.0 W/kg / normal";

SED = " 0.0 kJ/kg";

Coil Power = "15 %";

Max B1+rms = "0.91 uT";

PNS / level = "67 % / normal";

dB/dt = "68.5 T/s";

Sound Pressure Level (dB) = 16.7623653;

### A.2.3 B0 map

Patient weight [kg] = 5;

SmartSelect = "yes";

Coil 1 (exclude) = "None";

Uniformity = "CLEAR";

FOV AP (mm) = 224;

RL (mm) = 224;

FH (mm) = 14;

ACQ voxel size AP (mm) = 2;

RL (mm) = 2;

FH (mm) = 2;

Recon voxel size AP (mm) = 2;

RL (mm) = 2;

FH (mm) = 2;

Fold-over suppression = "no";

Slice oversampling = "default";

RF select. FOS = "no";

ENCASE enable = "no";

Reconstruction matrix = 112;

SENSE = "yes";

P reduction (RL) = 1;

S reduction (FH) = 1;

k-t BLAST = "no";

Stacks = 1;

slices = 7;

slice orientation = "transverse";

fold-over direction = "RL";

fat shift direction = "P";

Stack Offc. AP (P=+mm) = 1.76040018;

RL (L=+mm) = -0.822219431;

FH (H=+mm) = -0.297277153;

Ang. AP (deg) = 0;

RL (deg) = -0;

FH (deg) = -0;

Free rotatable = "no";

Multi-chunk = "no";

Large table movement = "no";

PlanAlign = "no";

REST slabs = 0;

Interactive positioning = "no";

Patient position = "head first";

Patient body position = "head first";

Patient orientation = "supine";

Patient body orientation = "supine";

Scan type = "Imaging";

Scan mode = "3D";

technique = "FFE";

loop order = "zy_order";

Contrast enhancement = "T1";

Acquisition mode = "cartesian";

Fast Imaging mode = "none";

3D non-selective = "no";

Echoes = 1;

partial echo = "no";

shifted echo = "no";

TE = "shortest";

Flip angle (deg) = 1;

TR = "shortest";

Halfscan = "no";

Water-fat shift = "maximum";

Shim = "manual";

Manual shim reset = "yes";

X shim value = 0;

Y shim value = 0;

Z shim value = 0;

Z2 shim value = 0;

ZX shim value = 0;

ZY shim value = 0;

X2-Y2 shim value = 0;

2XY shim value = 0;

mDIXON = "no";

Fat suppression = "no";

Water suppression = "no";

MTC = "no";

Research prepulse = "no";

Diffusion mode = "no";

Multi-transmit = "no";

SAR mode = "high";

B1 mode = "default";

SAR allow first level = "yes";

Patient pregnancy = "no";

Patient WB SAR [W/kg] = 0;

Patient Head SAR [W/kg] = 0;

Patient max. dB/dt [T/s] = 0;

Max slewrate [T/m/s] = 0;

Max. B1+rms [uT] = 0;

PNS mode = "high";

Gradient mode = "default";

SofTone mode = "no";

Cardiac synchronization = "no";

Heart rate > 250 bpm = "no";

Respiratory compensation = "no";

Navigator respiratory comp = "no";

Flow compensation = "no";

fMRI echo stabilisation = "no";

NSA = 1;

Angio / Contrast enh. = "no";

Quantitative flow = "no";

Manual start = "no";

Dynamic study = "no";

Arterial Spin labeling = "no";

Preparation phases = "full";

Interactive F0 = "no";

B0 field map = "yes";

field map technique = "multi-acquisition";

delta TE (ms) = 2.29999995;

B1 field map = "no";

MIP/MPR = "no";

SWIp = "no";

Images = "M", "P", (2) "no";

Autoview image = "M";

Calculated images = "B0", (3) "no";

Reference tissue = "Grey matter";

Recon compression = "No";

Preset window contrast = "soft";

Reconstruction mode = "real time";

Save raw data = "no";

Hardcopy protocol = "no";

Image filter = "no";

Uniformity correction = "no";

Geometry correction = "default";

Elliptical k-space shutter = "no";

IF_info_seperator = 0;

Research Options used = "28";

Total scan duration = "00:17.1";

Rel. SNR = 1;

Act. TR/TE (ms) = "8.4 / 3.4";

ACQ matrix M x P = "112 x 112";

ACQ voxel MPS (mm) = "2.00 / 2.00 / 2.00";

REC voxel MPS (mm) = "2.00 / 2.00 / 2.00";

Scan percentage (%) = 100;

Act. slice gap (mm) = 0;

Act. WFS (pix) / BW (Hz) = "1.136 / 382.2";

Min. WFS (pix) / Max. BW (Hz) = "0.245 / 1771.5";

Head SAR = " 0 %";

Whole body SAR / level = "0.0 W/kg / normal";

SED = " 0.0 kJ/kg";

Max B1+rms = "0.07 uT";

PNS / level = "38 % / normal";

dB/dt = "46.5 T/s";

Sound Pressure Level (dB) = 14.9578619;

### A.2.4 B1 map (left transmit channel)

Patient weight [kg] = 5;

SmartSelect = "yes";

Coil 1 (exclude) = "None";

Uniformity = "CLEAR";

FOV AP (mm) = 224;

RL (mm) = 224;

FH (mm) = 14;

ACQ voxel size AP (mm) = 2;

RL (mm) = 2;

FH (mm) = 2;

Recon voxel size AP (mm) = 2;

RL (mm) = 2;

FH (mm) = 2;

Fold-over suppression = "no";

Slice oversampling = "default";

RF select. FOS = "no";

ENCASE enable = "no";

Reconstruction matrix = 112;

SENSE = "yes";

P reduction (RL) = 1;

S reduction (FH) = 1;

k-t BLAST = "no";

Stacks = 1;

slices = 7;

slice orientation = "transverse";

fold-over direction = "RL";

fat shift direction = "P";

Stack Offc. AP (P=+mm) = 1.76040018;

RL (L=+mm) = -0.822219431;

FH (H=+mm) = -0.297277153;

Ang. AP (deg) = 0;

RL (deg) = -0;

FH (deg) = -0;

Free rotatable = "no";

Multi-chunk = "no";

Large table movement = "no";

PlanAlign = "no";

REST slabs = 0;

Interactive positioning = "no";

Patient position = "head first";

Patient body position = "head first";

Patient orientation = "supine";

Patient body orientation = "supine";

Scan type = "Imaging";

Scan mode = "3D";

technique = "FFE";

loop order = "zy_order";

Contrast enhancement = "T1";

Acquisition mode = "cartesian";

Fast Imaging mode = "none";

3D non-selective = "no";

Echoes = 1;

partial echo = "no";

shifted echo = "no";

TE = "user defined";

(ms) = 5;

Flip angle (deg) = 50;

TR = "user defined";

(ms) = 40;

Halfscan = "no";

Water-fat shift = "maximum";

RF Shims = "fixed";

Shim = "manual";

Manual shim reset = "yes";

X shim value = 0;

Y shim value = 0;

Z shim value = 0;

Z2 shim value = 0;

ZX shim value = 0;

ZY shim value = 0;

X2-Y2 shim value = 0;

2XY shim value = 0;

mDIXON = "no";

Fat suppression = "no";

Water suppression = "no";

MTC = "no";

Research prepulse = "no";

Diffusion mode = "no";

Multi-transmit = "yes";

Transmit channels = "left";

SAR mode = "high";

B1 mode = "default";

SAR allow first level = "yes";

Patient pregnancy = "no";

Patient WB SAR [W/kg] = 0;

Patient Head SAR [W/kg] = 0;

Patient max. dB/dt [T/s] = 0;

Max slewrate [T/m/s] = 0;

Max. B1+rms [uT] = 0;

PNS mode = "high";

Gradient mode = "default";

SofTone mode = "no";

Cardiac synchronization = "no";

Heart rate > 250 bpm = "no";

Respiratory compensation = "no";

Navigator respiratory comp = "no";

Flow compensation = "no";

fMRI echo stabilisation = "no";

NSA = 1;

Angio / Contrast enh. = "no";

Quantitative flow = "no";

Manual start = "no";

Dynamic study = "no";

Arterial Spin labeling = "no";

Preparation phases = "full";

Interactive F0 = "no";

B0 field map = "no";

B1 field map = "yes";

field map technique = "dual TR";

TR extension = 120;

selective = "yes";

grad spoil factor = 10;

MIP/MPR = "no";

SWIp = "no";

Images = "M", (3) "no";

Autoview image = "M";

Calculated images = "B1", (3) "no";

Reference tissue = "Grey matter";

Recon compression = "No";

Preset window contrast = "soft";

Reconstruction mode = "real time";

Save raw data = "no";

Hardcopy protocol = "no";

Image filter = "no";

Geometry correction = "default";

Elliptical k-space shutter = "no";

IF_info_seperator = 0;

Research Options used = "28,47";

Total scan duration = "03:22.4";

Rel. SNR = 1;

Act. TR/TE (ms) = "40 / 5.0";

ACQ matrix M x P = "112 x 112";

ACQ voxel MPS (mm) = "2.00 / 2.00 / 2.00";

REC voxel MPS (mm) = "2.00 / 2.00 / 2.00";

Scan percentage (%) = 100;

Act. slice gap (mm) = 0;

Act. WFS (pix) / BW (Hz) = "1.829 / 237.5";

Min. WFS (pix) / Max. BW (Hz) = "0.426 / 1019.2";

Min. TR/TE (ms) = "21 / 4.2";

Head SAR = "< 4 %";

Whole body SAR / level = "0.0 W/kg / normal";

SED = " 0.0 kJ/kg";

Coil Power = "4 %";

Max B1+rms = "0.45 uT";

PNS / level = "16 % / normal";

dB/dt = "14.7 T/s";

Sound Pressure Level (dB) = -5.84808111;

### A.2.5 B1 map (right transmit channel)

SmartSelect = "yes";

Coil 1 (exclude) = "None";

Uniformity = "CLEAR";

FOV AP (mm) = 224;

RL (mm) = 224;

FH (mm) = 14;

ACQ voxel size AP (mm) = 2;

RL (mm) = 2;

FH (mm) = 2;

Recon voxel size AP (mm) = 2;

RL (mm) = 2;

FH (mm) = 2;

Fold-over suppression = "no";

Slice oversampling = "default";

RF select. FOS = "no";

ENCASE enable = "no";

Reconstruction matrix = 112;

SENSE = "yes";

P reduction (RL) = 1;

S reduction (FH) = 1;

k-t BLAST = "no";

Stacks = 1;

slices = 7;

slice orientation = "transverse";

fold-over direction = "RL";

fat shift direction = "P";

Stack Offc. AP (P=+mm) = 1.76040018;

RL (L=+mm) = -0.822219431;

FH (H=+mm) = -0.297277153;

Ang. AP (deg) = 0;

RL (deg) = -0;

FH (deg) = -0;

Free rotatable = "no";

Multi-chunk = "no";

Large table movement = "no";

PlanAlign = "no";

REST slabs = 0;

Interactive positioning = "no";

Patient position = "head first";

Patient body position = "head first";

Patient orientation = "supine";

Patient body orientation = "supine";

Scan type = "Imaging";

Scan mode = "3D";

technique = "FFE";

loop order = "zy_order";

Contrast enhancement = "T1";

Acquisition mode = "cartesian";

Fast Imaging mode = "none";

3D non-selective = "no";

Echoes = 1;

partial echo = "no";

shifted echo = "no";

TE = "user defined";

(ms) = 5;

Flip angle (deg) = 50;

TR = "user defined";

(ms) = 40;

Halfscan = "no";

Water-fat shift = "maximum";

RF Shims = "fixed";

Shim = "manual";

Manual shim reset = "yes";

X shim value = 0;

Y shim value = 0;

Z shim value = 0;

Z2 shim value = 0;

ZX shim value = 0;

ZY shim value = 0;

X2-Y2 shim value = 0;

2XY shim value = 0;

mDIXON = "no";

Fat suppression = "no";

Water suppression = "no";

MTC = "no";

Research prepulse = "no";

Diffusion mode = "no";

Multi-transmit = "yes";

Transmit channels = "right";

SAR mode = "high";

B1 mode = "default";

SAR allow first level = "yes";

Patient pregnancy = "no";

Patient WB SAR [W/kg] = 0;

Patient Head SAR [W/kg] = 0;

Patient max. dB/dt [T/s] = 0;

Max slewrate [T/m/s] = 0;

Max. B1+rms [uT] = 0;

PNS mode = "high";

Gradient mode = "default";

SofTone mode = "no";

Cardiac synchronization = "no";

Heart rate > 250 bpm = "no";

Respiratory compensation = "no";

Navigator respiratory comp = "no";

Flow compensation = "no";

fMRI echo stabilisation = "no";

NSA = 1;

Angio / Contrast enh. = "no";

Quantitative flow = "no";

Manual start = "no";

Dynamic study = "no";

Arterial Spin labeling = "no";

Preparation phases = "full";

Interactive F0 = "no";

B0 field map = "no";

B1 field map = "yes";

field map technique = "dual TR";

TR extension = 120;

selective = "yes";

grad spoil factor = 10;

MIP/MPR = "no";

SWIp = "no";

Images = "M", (3) "no";

Autoview image = "M";

Calculated images = "B1", (3) "no";

Reference tissue = "Grey matter";

Recon compression = "No";

Preset window contrast = "soft";

Reconstruction mode = "real time";

Save raw data = "no";

Hardcopy protocol = "no";

Image filter = "no";

Geometry correction = "default";

Elliptical k-space shutter = "no";

IF_info_seperator = 0;

Research Options used = "28,47";

Total scan duration = "03:22.4";

Rel. SNR = 1;

Act. TR/TE (ms) = "40 / 5.0";

ACQ matrix M x P = "112 x 112";

ACQ voxel MPS (mm) = "2.00 / 2.00 / 2.00";

REC voxel MPS (mm) = "2.00 / 2.00 / 2.00";

Scan percentage (%) = 100;

Act. slice gap (mm) = 0;

Act. WFS (pix) / BW (Hz) = "1.829 / 237.5";

Min. WFS (pix) / Max. BW (Hz) = "0.426 / 1019.2";

Min. TR/TE (ms) = "21 / 4.2";

Head SAR = "< 4 %";

Whole body SAR / level = "0.0 W/kg / normal";

SED = " 0.0 kJ/kg";

Coil Power = "4 %";

Max B1+rms = "0.45 uT";

PNS / level = "16 % / normal";

dB/dt = "14.7 T/s";

Sound Pressure Level (dB) = -5.84808111;

### A.2.6 B1 map

SmartSelect = "yes";

Coil 1 (exclude) = "None";

Uniformity = "CLEAR";

FOV AP (mm) = 224;

RL (mm) = 224;

FH (mm) = 14;

ACQ voxel size AP (mm) = 2;

RL (mm) = 2;

FH (mm) = 2;

Recon voxel size AP (mm) = 2;

RL (mm) = 2;

FH (mm) = 2;

Fold-over suppression = "no";

Slice oversampling = "default";

RF select. FOS = "no";

ENCASE enable = "no";

Reconstruction matrix = 112;

SENSE = "yes";

P reduction (RL) = 1;

S reduction (FH) = 1;

k-t BLAST = "no";

Stacks = 1;

slices = 7;

slice orientation = "transverse";

fold-over direction = "RL";

fat shift direction = "P";

Stack Offc. AP (P=+mm) = 1.76040018;

RL (L=+mm) = -0.822219431;

FH (H=+mm) = -0.297277153;

Ang. AP (deg) = 0;

RL (deg) = -0;

FH (deg) = -0;

Free rotatable = "no";

Multi-chunk = "no";

Large table movement = "no";

PlanAlign = "no";

REST slabs = 0;

Interactive positioning = "no";

Patient position = "head first";

Patient body position = "head first";

Patient orientation = "supine";

Patient body orientation = "supine";

Scan type = "Imaging";

Scan mode = "3D";

technique = "FFE";

loop order = "zy_order";

Contrast enhancement = "T1";

Acquisition mode = "cartesian";

Fast Imaging mode = "none";

3D non-selective = "no";

Echoes = 1;

partial echo = "no";

shifted echo = "no";

TE = "user defined";

(ms) = 5;

Flip angle (deg) = 50;

TR = "user defined";

(ms) = 40;

Halfscan = "no";

Water-fat shift = "maximum";

Shim = "manual";

Manual shim reset = "yes";

X shim value = 0;

Y shim value = 0;

Z shim value = 0;

Z2 shim value = 0;

ZX shim value = 0;

ZY shim value = 0;

X2-Y2 shim value = 0;

2XY shim value = 0;

mDIXON = "no";

Fat suppression = "no";

Water suppression = "no";

MTC = "no";

Research prepulse = "no";

Diffusion mode = "no";

Multi-transmit = "no";

SAR mode = "high";

B1 mode = "default";

SAR allow first level = "yes";

Patient pregnancy = "no";

Patient WB SAR [W/kg] = 0;

Patient Head SAR [W/kg] = 0;

Patient max. dB/dt [T/s] = 0;

Max slewrate [T/m/s] = 0;

Max. B1+rms [uT] = 0;

PNS mode = "high";

Gradient mode = "default";

SofTone mode = "no";

Cardiac synchronization = "no";

Heart rate > 250 bpm = "no";

Respiratory compensation = "no";

Navigator respiratory comp = "no";

Flow compensation = "no";

fMRI echo stabilisation = "no";

NSA = 1;

Angio / Contrast enh. = "no";

Quantitative flow = "no";

Manual start = "no";

Dynamic study = "no";

Arterial Spin labeling = "no";

Preparation phases = "full";

Interactive F0 = "no";

B0 field map = "no";

B1 field map = "yes";

field map technique = "dual TR";

TR extension = 120;

selective = "yes";

grad spoil factor = 10;

MIP/MPR = "no";

SWIp = "no";

Images = "M", (3) "no";

Autoview image = "M";

Calculated images = "B1", (3) "no";

Reference tissue = "Grey matter";

Recon compression = "No";

Preset window contrast = "soft";

Reconstruction mode = "real time";

Save raw data = "no";

Hardcopy protocol = "no";

Image filter = "no";

Geometry correction = "default";

Elliptical k-space shutter = "no";

IF_info_seperator = 0;

Research Options used = "28,47";

Total scan duration = "03:22.4";

Rel. SNR = 1;

Act. TR/TE (ms) = "40 / 5.0";

ACQ matrix M x P = "112 x 112";

ACQ voxel MPS (mm) = "2.00 / 2.00 / 2.00";

REC voxel MPS (mm) = "2.00 / 2.00 / 2.00";

Scan percentage (%) = 100;

Act. slice gap (mm) = 0;

Act. WFS (pix) / BW (Hz) = "2.288 / 189.8";

Min. WFS (pix) / Max. BW (Hz) = "0.426 / 1019.2";

Min. TR/TE (ms) = "20 / 3.7";

Head SAR = "< 7 %";

Whole body SAR / level = "0.0 W/kg / normal";

SED = " 0.0 kJ/kg";

Max B1+rms = "0.63 uT";

PNS / level = "13 % / normal";

dB/dt = "16.2 T/s";

Sound Pressure Level (dB) = -8.5160799;

### A.2.7 Single slice FFE, single shot EPI read-out

SmartSelect = "yes";

Coil 1 (exclude) = "None";

Uniformity = "Classic";

FOV RL (mm) = 200;

AP (mm) = 200;

FH (mm) = 2;

ACQ voxel size RL (mm) = 2;

AP (mm) = 2;

Slice thickness (mm) = 2;

Recon voxel size RL (mm) = 1.78571427;

AP (mm) = 1.78571427;

Fold-over suppression = "no";

Reconstruction matrix = 112;

SENSE = "no";

MB SENSE = "no";

k-t BLAST = "no";

Stacks = 1;

type = "parallel";

slices = 1;

slice gap = "user defined";

gap (mm) = 0;

slice orientation = "transverse";

fold-over direction = "AP";

fat shift direction = "P";

Stack Offc. AP (P=+mm) = 1.76040018;

RL (L=+mm) = -0.822219431;

FH (H=+mm) = -0.297277153;

Ang. AP (deg) = 0;

RL (deg) = -0;

FH (deg) = -0;

Free rotatable = "no";

Minimum number of packages = 1;

Slice scan order = "HF";

Large table movement = "no";

PlanAlign = "no";

REST slabs = 0;

Interactive positioning = "no";

Patient position = "head first";

Patient body position = "head first";

Patient orientation = "supine";

Patient body orientation = "supine";

Scan type = "Imaging";

Scan mode = "MS";

technique = "FFE";

Contrast enhancement = "no";

Acquisition mode = "cartesian";

Fast Imaging mode = "EPI";

shot mode = "single-shot";

Echoes = 1;

partial echo = "no";

shifted echo = "no";

TE = "shortest";

Flip angle (deg) = 25;

TR = "user defined";

(ms) = 150;

Halfscan = "no";

Water-fat shift = "minimum";

Shim = "manual";

Manual shim reset = "yes";

X shim value = 0;

Y shim value = 0;

Z shim value = 0;

Z2 shim value = 0;

ZX shim value = 0;

ZY shim value = 0;

X2-Y2 shim value = 0;

2XY shim value = 0;

mDIXON = "no";

Fat suppression = "no";

Water suppression = "no";

MTC = "no";

Research prepulse = "no";

Diffusion mode = "no";

Multi-transmit = "no";

SAR mode = "high";

B1 mode = "default";

SAR allow first level = "yes";

Patient pregnancy = "no";

Patient WB SAR [W/kg] = 0;

Patient Head SAR [W/kg] = 0;

Patient max. dB/dt [T/s] = 0;

Max slewrate [T/m/s] = 0;

Max. B1+rms [uT] = 0;

PNS mode = "high";

Gradient mode = "maximum";

SofTone mode = "no";

Cardiac synchronization = "no";

Heart rate > 250 bpm = "no";

Respiratory compensation = "no";

Navigator respiratory comp = "no";

Flow compensation = "no";

Temporal slice spacing = "equidistant";

fMRI echo stabilisation = "no";

NSA = 1;

Angio / Contrast enh. = "no";

Quantitative flow = "no";

Manual start = "no";

Dynamic study = "individual";

dyn scans = 2001;

dyn scan times = "shortest";

fov time mode = "default";

dummy scans = 30;

immediate subtraction = "no";

fast next scan = "no";

synch. ext. device = "yes";

start at dyn. = 1;

interval (dyn) = 1;

dyn stabilization = "no";

prospect. motion corr. = "no";

Keyhole = "no";

Arterial Spin labeling = "no";

Preparation phases = "full";

Interactive F0 = "no";

B0 field map = "no";

B1 field map = "no";

MIP/MPR = "no";

SWIp = "no";

Images = "M", (3) "no";

Autoview image = "M";

Calculated images = (4) "no";

Reference tissue = "Grey matter";

Recon compression = "No";

Preset window contrast = "soft";

Reconstruction mode = "real time";

reuse memory = "no";

Save raw data = "no";

Hardcopy protocol = "no";

Image filter = "no";

Uniformity correction = "no";

Geometry correction = "default";

IF_info_seperator = 0;

Research Options used = "28";

Total scan duration = "05:05.4";

Rel. SNR = 1;

Act. TR/TE (ms) = "150 / 32";

Dyn. scan time = "0.150";

Time to k0 = "0.075";

ACQ matrix M x P = "100 x 99";

ACQ voxel MPS (mm) = "2.00 / 2.02 / 2.00";

REC voxel MPS (mm) = "1.79 / 1.79 / 2.00";

Scan percentage (%) = 99;

Packages = 1;

Min. slice gap (mm) = -0;

EPI factor = 99;

Act. WFS (pix) / BW (Hz) = "26.773 / 16.2";

BW in EPI freq. dir. (Hz) = "2258.8";

Min. WFS (pix) / Max. BW (Hz) = "24.475 / 17.7";

Min. TR/TE (ms) = "66 / 32";

Head SAR = "< 1 %";

Whole body SAR / level = "0.0 W/kg / normal";

SED = " 0.0 kJ/kg";

Max B1+rms = "0.19 uT";

PNS / level = "88 % / 1st level";

dB/dt = "70.9 T/s";

Sound Pressure Level (dB) = 19.4487247;
