# Supplementary material for: The YOUth cohort study: MRI protocol and test-retest reliability in adults
Source: Dev Cogn Neurosci. 2020 Jul 8;45:100816. doi: 10.1016/j.dcn.2020.100816 (PMC7365929; doi:10.1016/j.dcn.2020.100816)
Supplement: Supplementary file 2 [file mmc2.pdf]

Example of quality control output that is generated in YOUth after each session

Examples are obtained from different reports and intended just as illustration.
Participant identifiers and test dates are masked.

Quality control of T1-weighted scans

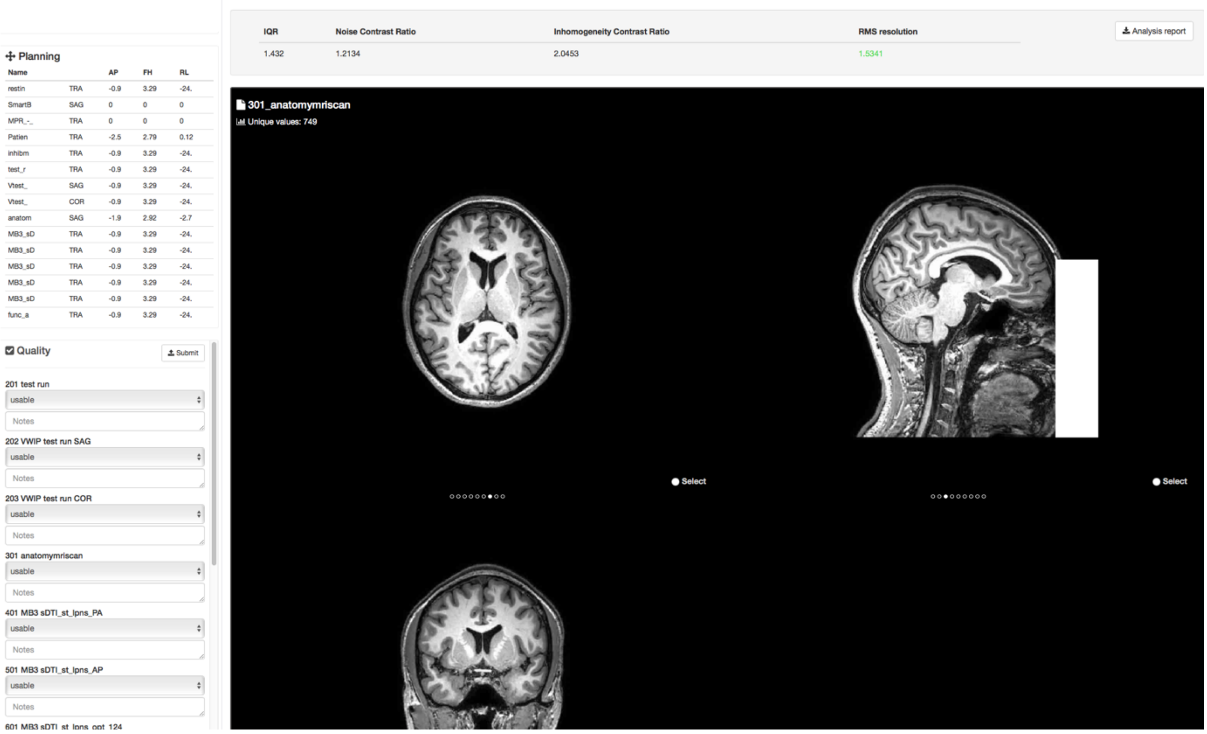

Quality control of Functional MRI scans

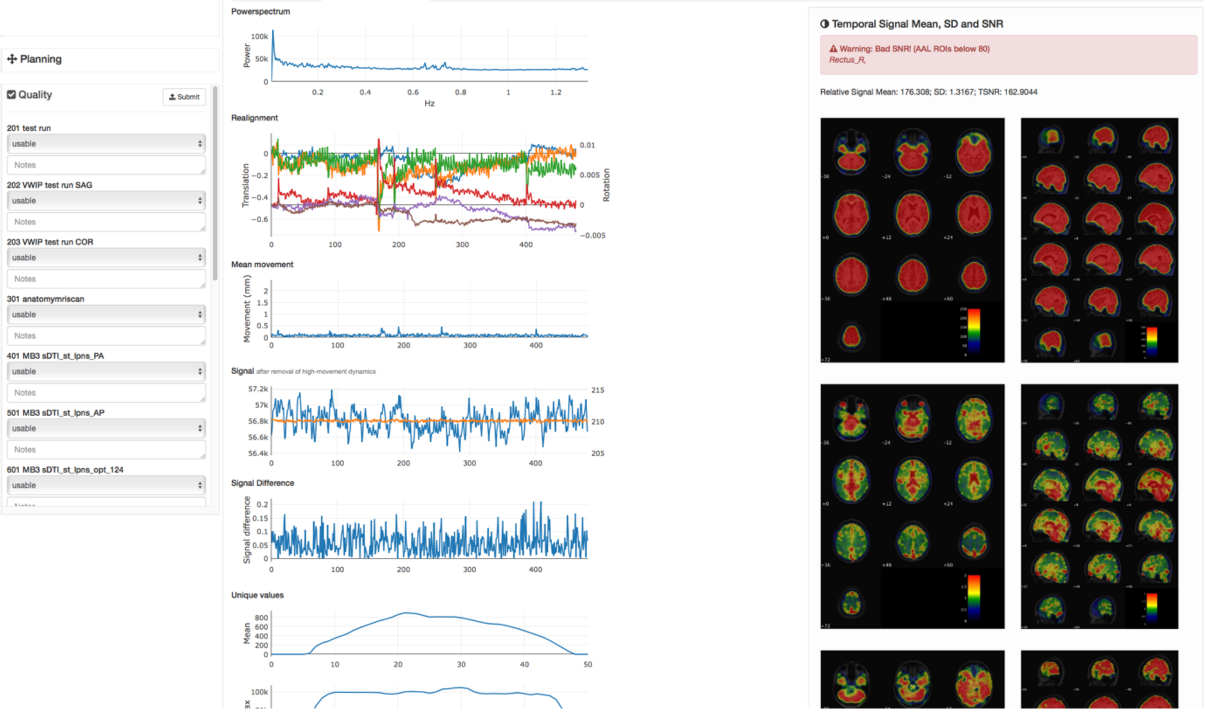

Quality control of DWI-scans

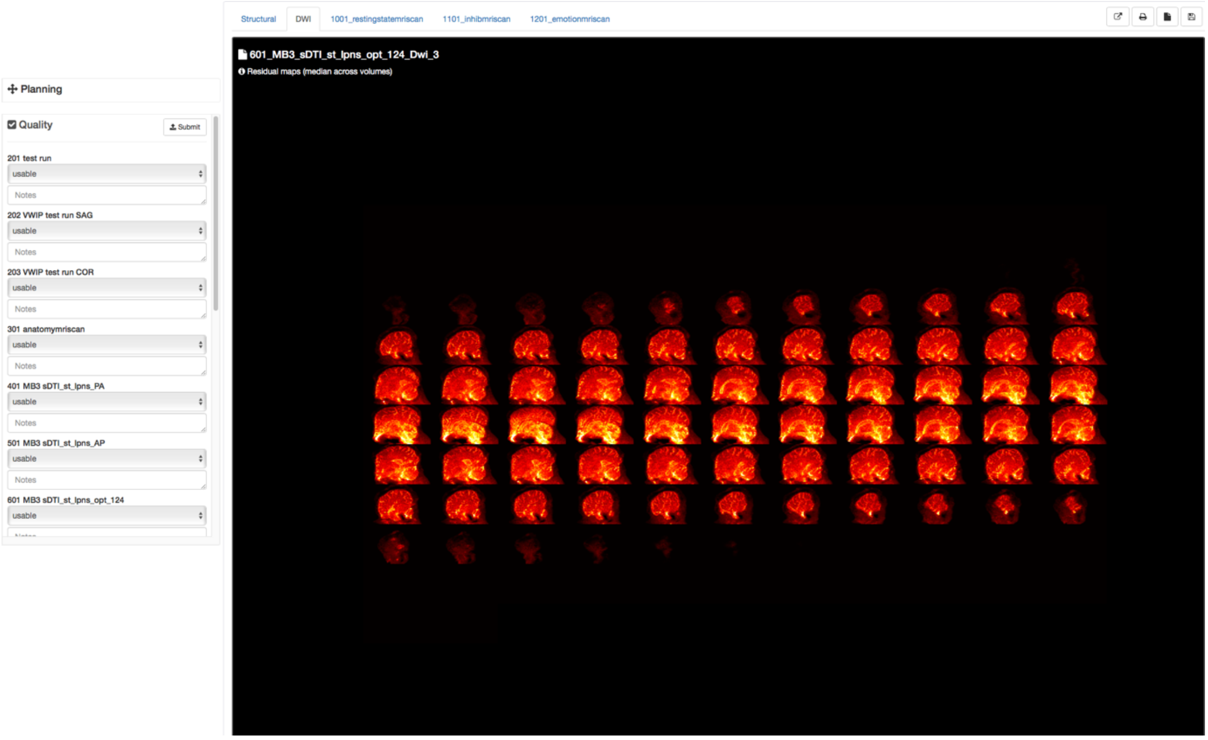

Including the Eddy quad report:

Single subject QC report generated using eddy quad v1.0.2

When using eddy and its QC tools, we ask you to please reference the papers describing the different aspects of the modelling and corrections. The following suggestion for a methods section and list of references has been tailored for you based on your eddy command line.

METHODS
The susceptibility induced off-resonance field was estimated from spin-echo EPI images acquired with different phase-encode directions (Andersson et al., 2003). This field was passed to "eddy", a tool that combined it with estimating gross subject movement and eddy current-induced distortions (Andersson & Sotiropoulos, 2016).The quality of the dataset was assessed using the eddy QC tools (Bastiani et al., 2019). Slices with signal loss caused by subject movement coinciding with the diffusion encoding were detected and replaced by predictions made by a Gaussian Process (Andersson et al., 2016).

REFERENCES
Jesper L.R. Andersson, Stefan Skare and John Ashburner. 2003. How to correct susceptibility distortions in spin-echo echo-planar images: application to diffusion tensor imaging. NeuroImage 20:870-888

Jesper L.R. Andersson and Stamatios N. Sotiropoulos. 2016. An integrated approach to correction for off-resonance effects and subject movement in diffusion MR imaging. NeuroImage 125:1063-1078

Matteo Bastiani, Michiel Cottaar, Sean P. Fitzgibbon, Sana Suri, Fidel Alfaro-Almagro, Stamatios N. Sotiropoulos, Saad Jbabdi and Jesper L.R. Andersson. 2019. Automated quality control for within and between studies diffusion MRI data using a non-parametric framework for movement and distortion correction. NeuroImage 184:801-812

Jesper L.R. Andersson, Mark S. Graham, Eniko Zsoldos and Stamatios N. Sotiropoulos. 2016. Incorporating outlier detection and replacement into a non-parametric framework for movement and distortion correction of diffusion MR images. NeuroImage 141:556-572

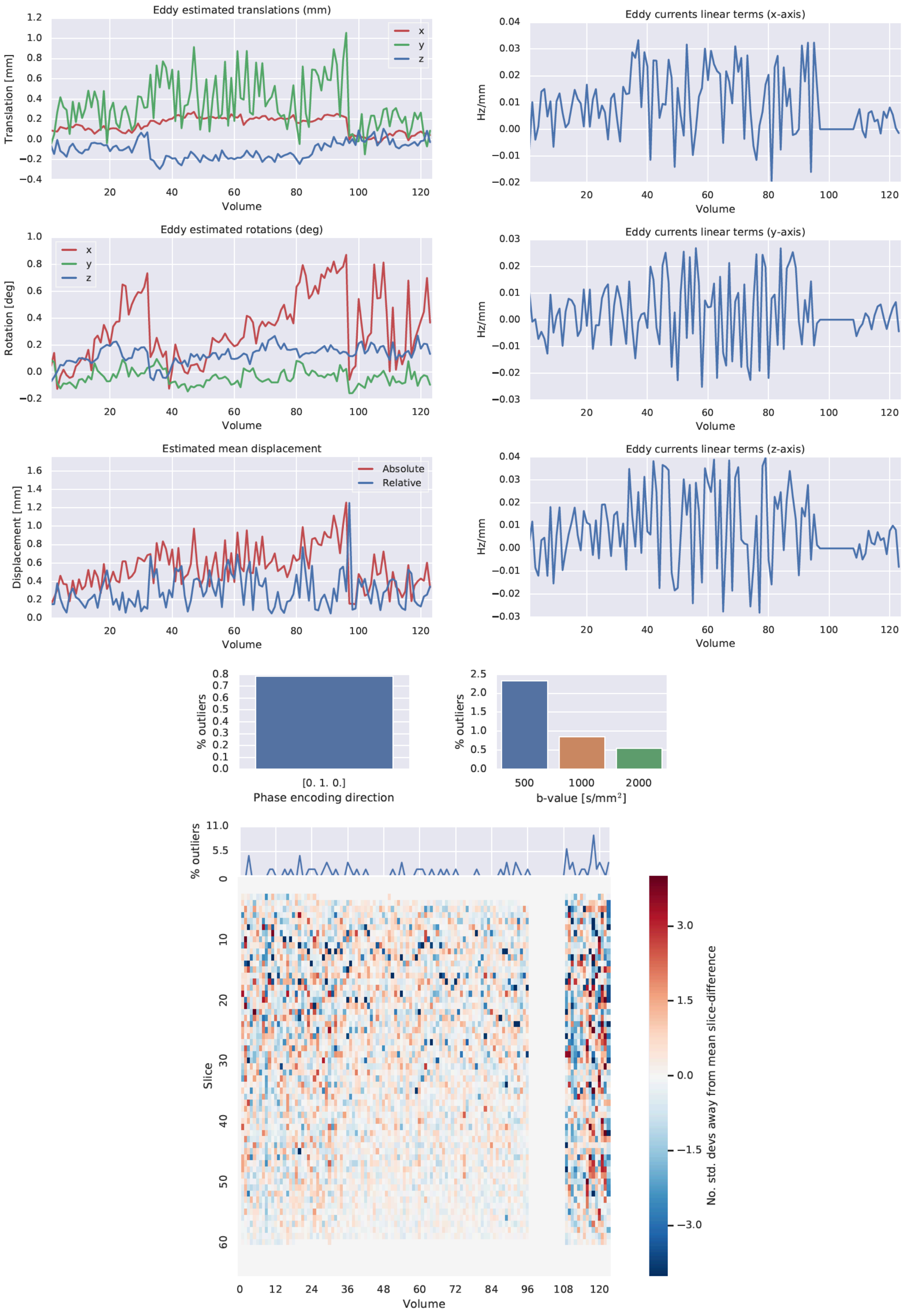

Eddy estimated translations (mm)
Translation [mm]
Volume
x
y
z
Eddy currents linear terms (x-axis)
Hz/mm
Volume
Eddy estimated rotations (deg)
Rotation [deg]
Volume
Eddy currents linear terms (y-axis)
Estimated mean displacement
Displacement [mm]
Absolute
Relative
Eddy currents linear terms (z-axis)
% outliers
[0. 1. 0.]
Phase encoding direction
500
1000
2000
b-value [s/mm^2]
Slice
No. std. devs away from mean slice-difference

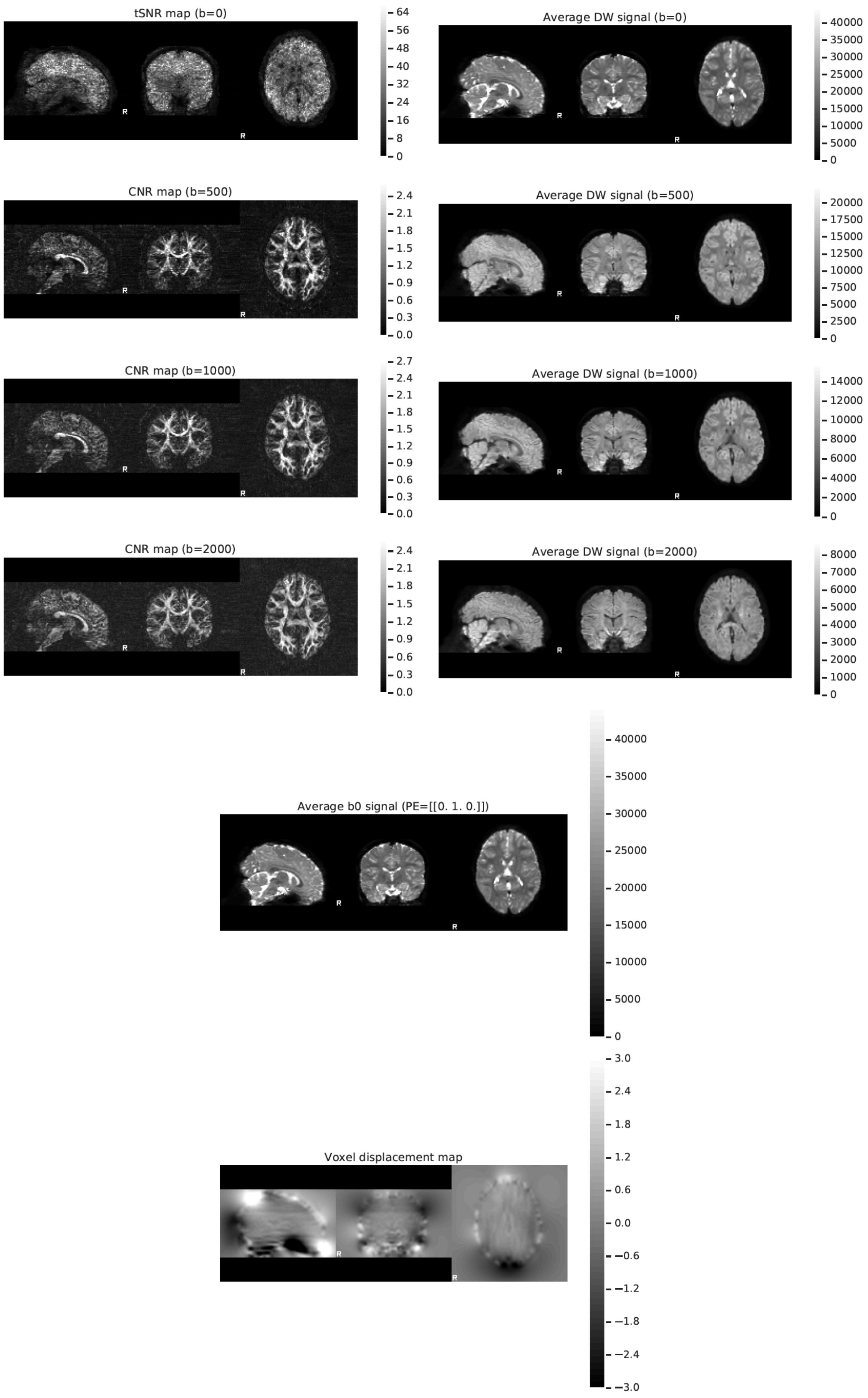

tSNR map (b=0)
Average DW signal (b=0)
CNR map (b=500)
Average DW signal (b=500)
CNR map (b=1000)
Average DW signal (b=1000)
CNR map (b=2000)
Average DW signal (b=2000)
Average b0 signal (PE=[[0. 1. 0.]])
Voxel displacement map
