# Supplementary material for: The YOUth cohort study: MRI protocol and test-retest reliability in adults
Source: Dev Cogn Neurosci. 2020 Jul 8;45:100816. doi: 10.1016/j.dcn.2020.100816 (PMC7365929; doi:10.1016/j.dcn.2020.100816)
Supplement: Supplementary file 3 [file mmc3.docx]

**The YOUth cohort study: MRI protocol and test-retest reliability in adults -** *Supplement C*

Elizabeth E.L. Buimer*^1^, Pascal Pas*^1^, Rachel M. Brouwer^1^, Martijn Froeling^2^, Hans Hoogduin^2^, Alexander Leemans^2^, Peter Luijten^3^, Bastiaan J. van Nierop^2^, Mathijs Raemaekers^1^, Hugo G. Schnack^1^, Jalmar Teeuw^1^, Matthijs Vink^1,4^, Fredy Visser^5^, Hilleke E. Hulshoff Pol^1^ and René C.W. Mandl^1^

** Elizabeth Buimer and Pascal Pas contributed equally*

^1.^ UMCU Brain Center, University Medical Center Utrecht, University Utrecht, Utrecht, The Netherlands

^2.^ Image Sciences Institute, University Medical Center Utrecht and Utrecht University, Utrecht, the Netherlands.

^3.^ Department of Radiology, University Medical Center Utrecht, Utrecht, The Netherlands

^4.^ Department of Psychology, Utrecht University, Utrecht, The Netherlands

^5.^ Philips Healthcare, Best, The Netherlands

**Corresponding author**

René C. W. Mandl, Utrecht Brain Center, University Medical Center Utrecht, Department of Psychiatry

Heidelberglaan 100 (Room A01.126), 3584CX Utrecht, The Netherlands, +31-0887559705, r.m.mandl@umcutrecht.nl


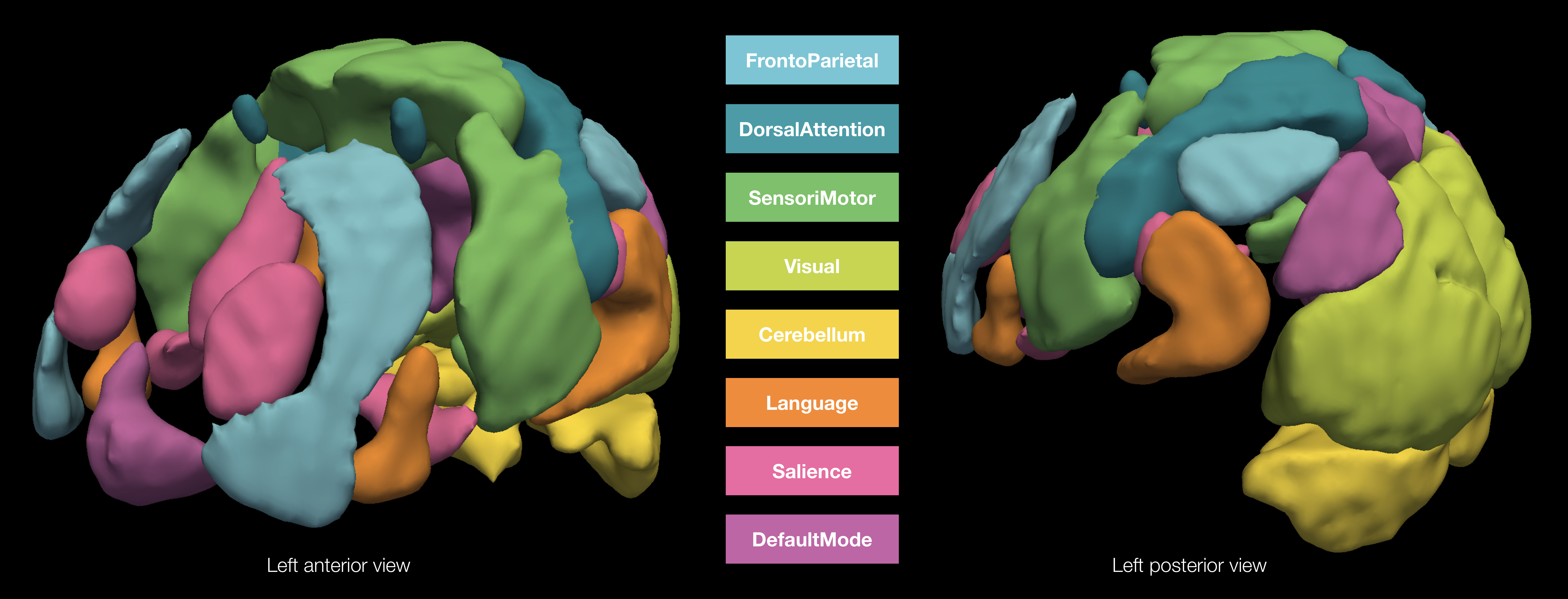


Figure S1. Atlas of canonical resting-state networks and their spatially distinct components provided by the CONN toolbox version 18.a based on ICA decomposition of 497 young adults from the Human Connectome Project.

**Table S1. Structural T1-weighted test-retest reliability for ROIs of the Desikan-Killiany atlas.**

|  | **ICC [95% CI]** | | | | | | |
| --- | --- | --- | --- | --- | --- | --- | --- |
|  | Volume | | Surface area | | Thickness | | |
| **Subcortical region** | *Left hemisphere* | *Right hemisphere* |  | | | | |
| thalamus | 0.97 [0.90 to 0.99] | 0.97 [0.91 to 0.99] |  |  |  |  |  |
| caudatus | 0.96 [0.89 to 0.99] | 0.97 [0.92 to 0.99] |  |  |  |  |  |
| putamen | 0.97 [0.91 to 0.99] | 0.99 [0.98 to 1.00] |  |  |  |  |  |
| pallidum | 0.90 [0.73 to 0.96] | 0.94 [0.83 to 0.98] |  |  |  |  |  |
| hippocampus | 0.97 [0.93 to 0.99] | 0.97 [0.90 to 0.99] |  |  |  |  |  |
| amygdala | 0.93 [0.80 to 0.97] | 0.86 [0.66 to 0.95] |  |  |  |  |  |
| accumbens | 0.85^++^ [0.62 to 0.94] | 0.86 [0.65 to 0.95] |  |  |  |  |  |
| **Cortical region** | *Left hemisphere* | *Right hemisphere* | *Left hemisphere* | *Right hemisphere* | | *Left hemisphere* | *Right hemisphere* |
| banks superior temporal sulcus | 0.95 [0.87 to 0.98] | 0.94 [0.85 to 0.98] | 0.98 [0.95 to 0.99] | 0.98 [0.94 to 0.99] | | 0.82 [0.56 to 0.94] | 0.92 [0.79 to 0.97] |
| caudal anterior cingulate cortex | 0.97 [0.89 to 0.99] | 0.91 [0.75 to 0.97] | 0.98 [0.96 to 0.99] | 0.96 [0.89 to 0.99] | | 0.67 [0.28 to 0.87] | 0.80 [0.50 to 0.92] |
| caudal middle frontal gyrus | 0.98 [0.94 to 0.99] | 0.96 [0.88 to 0.98] | 0.99 [0.98 to 1.00] | 0.99 [0.96 to 1.00] | | 0.83 [0.59 to 0.94] | 0.60 [0.18 to 0.84] |
| cuneus | 0.97 [0.92 to 0.99] | 0.98 [0.96 to 0.99] | 0.98 [0.95 to 0.99] | 0.99 [0.97 to 1.00] | | 0.88 [0.69 to 0.96] | 0.94 [0.84 to 0.98] |
| entorhinal cortex | 0.81 [0.53 to 0.93] | 0.71 [0.34 to 0.89] | 0.86 [0.63 to 0.95] | 0.90 [0.74 to 0.96] | | 0.90 [0.75 to 0.96] | 0.76 [0.44 to 0.91] |
| fusiform gyrus | 0.96 [0.90 to 0.99] | 0.96 [0.89 to 0.99] | 0.99 [0.97 to 1.00] | 0.99 [0.99 to 1.00] | | 0.89 [0.73 to 0.96] | 0.83 [0.59 to 0.94] |
| inferior parietal cortex | 0.99 [0.96 to 1.00] | 0.98 [0.94 to 0.99] | 0.99 [0.98 to 1.00] | 0.99 [0.98 to 1.00] | | 0.82 [0.55 to 0.93] | 0.80 [0.50 to 0.92] |
| inferior temporal gyrus | 0.95 [0.87 to 0.98] | 0.93 [0.81 to 0.97] | 0.99 [0.97 to 1.00] | 0.98 [0.93 to 0.99] | | 0.86 [0.66 to 0.95] | 0.84 [0.59 to 0.94] |
| isthmus cingulate cortex | 0.96 [0.89 to 0.99] | 0.94 [0.84 to 0.98] | 0.97 [0.92 to 0.99] | 0.99 [0.96 to 0.99] | | 0.89 [0.71 to 0.96] | 0.87 [0.67 to 0.95] |
| lateral occipital cortex | 1.00 [0.99 to 1.00] | 0.99 [0.98 to 1.00] | 1.00 [0.99 to 1.00] | 1.00 [0.99 to 1.00] | | 0.96 [0.89 to 0.99] | 0.90 [0.73 to 0.96] |
| lateral orbitofrontal cortex | 0.95 [0.86 to 0.98] | 0.92 [0.79 to 0.97] | 0.96 [0.89 to 0.99] | 0.89 [0.72 to 0.96] | | 0.85 [0.63 to 0.94] | 0.67 [0.27 to 0.87] |
| lingual gyrus | 0.99 [0.96 to 1.00] | 0.98 [0.95 to 0.99] | 0.98 [0.95 to 0.99] | 0.99 [0.98 to 1.00] | | 0.82 [0.57 to 0.93] | 0.82 [0.57 to 0.93] |
| medial orbitofrontal cortex | 0.89 [0.71 to 0.96] | 0.96 [0.90 to 0.99] | 0.86 [0.65 to 0.95] | 0.94 [0.84 to 0.98] | | 0.65 [0.27 to 0.86] | 0.51 [0.04 to 0.80] |
| middle temporal gyrus | 0.96 [0.89 to 0.99] | 0.98 [0.93 to 0.99] | 0.98 [0.94 to 0.99] | 0.99 [0.98 to 1.00] | | 0.84 [0.60 to 0.94] | 0.90 [0.73 to 0.96] |
| parahippocampal gyrus | 0.96 [0.88 to 0.98] | 0.99 [0.96 to 0.99] | 0.98 [0.94 to 0.99] | 0.98 [0.94 to 0.99] | | 0.97 [0.92 to 0.99] | 0.85 [0.64 to 0.95] |
| paracentral lobule | 0.99 [0.96 to 0.99] | 0.95 [0.88 to 0.98] | 0.99 [0.98 to 1.00] | 0.97 [0.93 to 0.99] | | 0.89 [0.71 to 0.96] | 0.85 [0.63 to 0.94] |
| pars opercularis | 0.97 [0.89 to 0.99] | 0.98 [0.94 to 0.99] | 0.98 [0.94 to 0.99] | 0.98 [0.96 to 0.99] | | 0.88 [0.69 to 0.96] | 0.90 [0.74 to 0.96] |
| pars orbitalis | 0.95 [0.86 to 0.98] | 0.95 [0.88 to 0.98] | 0.96 [0.88 to 0.98] | 0.97 [0.90 to 0.99] | | 0.85 [0.62 to 0.94] | 0.88 [0.69 to 0.95] |
| pars triangularis | 0.98 [0.94 to 0.99] | 0.96 [0.89 to 0.99] | 0.99 [0.98 to 1.00] | 0.99 [0.98 to 1.00] | | 0.91 [0.78 to 0.97] | 0.72 [0.36 to 0.89] |
| pericalcarine cortex | 0.97 [0.90 to 0.99] | 0.97 [0.91 to 0.99] | 0.99 [0.97 to 1.00] | 0.99 [0.97 to 1.00] | | 0.72 [0.36 to 0.89] | 0.87 [0.68 to 0.95] |
| postcentral gyrus | 0.98 [0.95 to 0.99] | 0.95 [0.87 to 0.98] | 0.99 [0.98 to 1.00] | 0.98 [0.95 to 0.99] | | 0.89 [0.71 to 0.96] | 0.91 [0.76 to 0.97] |
| posterior cingulate cortex | 0.99 [0.97 to 1.00] | 0.99 [0.97 to 1.00] | 0.99 [0.96 to 1.00] | 0.99 [0.96 to 1.00] | | 0.86 [0.64 to 0.95] | 0.90 [0.73 to 0.96] |
| precentral gyrus | 0.99 [0.96 to 0.99] | 0.97 [0.90 to 0.99] | 0.99 [0.98 to 1.00] | 0.99 [0.99 to 1.00] | | 0.94 [0.84 to 0.98] | 0.76 [0.44 to 0.91] |
| precuneus | 0.98 [0.92 to 0.99] | 0.97 [0.92 to 0.99] | 0.99 [0.96 to 1.00] | 0.99 [0.98 to 1.00] | | 0.88 [0.69 to 0.96] | 0.84 [0.60 to 0.94] |
| rostral anterior cingulate cortex | 0.97 [0.92 to 0.99] | 0.96 [0.89 to 0.99] | 0.98 [0.94 to 0.99] | 0.99 [0.97 to 1.00] | | 0.88 [0.69 to 0.96] | 0.87 [0.67 to 0.95] |
| rostral middle frontal gyrus | 0.98 [0.94 to 0.99] | 0.94 [0.83 to 0.98] | 0.98 [0.95 to 0.99] | 0.99 [0.97 to 1.00] | | 0.89 [0.72 to 0.96] | 0.07^++^ [ to 0.42 to 0.53] |
| superior frontal gyrus | 0.95 [0.87 to 0.98] | 0.96 [0.88 to 0.98] | 0.99 [0.98 to 1.00] | 1.00 [0.99 to 1.00] | | 0.71 [0.34 to 0.89] | 0.62 [0.22 to 0.85] |
| superior parietal cortex | 0.99 [0.96 to 1.00] | 0.98 [0.94 to 0.99] | 1.00 [0.99 to 1.00] | 0.99 [0.97 to 1.00] | | 0.84 [0.60 to 0.94] | 0.84 [0.60 to 0.94] |
| superior temporal gyrus | 0.96 [0.89 to 0.99] | 0.96 [0.88 to 0.98] | 0.99 [0.97 to 1.00] | 1.00 [0.99 to 1.00] | | 0.78 [0.47 to 0.92] | 0.89 [0.72 to 0.96] |
| supramarginal gyrus | 0.98 [0.95 to 0.99] | 0.99 [0.97 to 1.00] | 0.99 [0.97 to 1.00] | 1.00 [0.99 to 1.00] | | 0.74 [0.40 to 0.90] | 0.83 [0.59 to 0.94] |
| frontal pole | 0.73 [0.37 to 0.90] | 0.89 [0.72 to 0.96] | 0.53^++^ [0.09 to 0.80] | 0.91 [0.77 to 0.97] | | 0.88 [0.69 to 0.96] | 0.48 [0.03 to 0.78] |
| temporal pole | 0.65**^++^** [0.26 to 0.86] | 0.73 [0.37 to 0.90] | 0.74 [0.40 to 0.90] | 0.80 [0.53 to 0.92] | | 0.81 [0.54 to 0.93] | 0.60 [0.19 to 0.84] |
| transverse temporal gyrus | 0.94 [0.85 to 0.98] | 0.95 [0.87 to 0.98] | 0.95 [0.86 to 0.98] | 0.95 [0.87 to 0.98] | | 0.83 [0.58 to 0.94] | 0.91 [0.77 to 0.97] |
| insula | 0.83 [0.58 to 0.94] | 0.93 [0.80 to 0.97] | 0.70 [0.32 to 0.88] | 0.88 [0.70 to 0.96] | | 0.69 [0.30 to 0.88] | 0.67 [0.28 to 0.87] |

Abbreviations: ICC = intraclass correlation coefficient; CI = confidence interval; **^++^** = minimum ICC for (sub)cortical volume, cortical thickness or cortical surface area.

**Table S2. DWI Test-retest results for ROIs JHU Atlas.** ICC for FA and MD computed for each of the 48 regions of interest. CC-SNR is the Pearson correlation between the percentage difference (PD) computed for the SNR from the quality check and the PD computed for the FA or MD (with the level of significance between parentheses). CC-RM is the correlation between PD of the relative motion and the PD from the FA or MD. CC-MVD is the correlation mean voxel displacement (from the susceptibility correction in FSL’s eddy) and the PD from FA or MD.

| Region | Fractional anisotropy | | | | | | | | Mean diffusivity | | | | | | |
| --- | --- | --- | --- | --- | --- | --- | --- | --- | --- | --- | --- | --- | --- | --- | --- |
|  | **ICC** | **CC-SNR** | | **CC-RM** | | **CC-MVD** | | **ICC** | | **CC-SNR** | | **CC-RM** | | **CC-MVD** | |
| Middle cerebellar peduncle | 0.61 | 0.35 | (0.20) | -0.10 | (0.71) | -0.36 | (0.19) | 0.57 | | 0.12 | (0.66) | -0.07 | (0.81) | 0.06 | (0.83) |
| Pontine crossing tract | 0.51^++^ | 0.09 | (0.76) | 0.02 | (0.94) | -0.21 | (0.45) | 0.53 | | -0.04 | (0.88) | -0.18 | (0.53) | 0.04 | (0.88) |
| Genu of corpus callosum | 0.87 | 0.31 | (0.25) | -0.30 | (0.28) | 0.07 | (0.82) | 0.51 | | 0.11 | (0.69) | 0.44 | (0.10) | -0.46 | (0.09) |
| Body of corpus callosum | 0.89 | 0.45 | (0.09) | -0.56 | (0.03)* | -0.11 | (0.7) | 0.88 | | 0.49 | (0.06) | 0.22 | (0.43) | -0.11 | (0.69) |
| Splenium of corpus callosum | 0.71 | 0.34 | (0.22) | 0.58 | (0.02)* | -0.03 | (0.91) | 0.91 | | -0.28 | (0.32) | 0.09 | (0.76) | 0.32 | (0.25) |
| Fornix (column and body of fornix) | 0.90 | 0.43 | (0.11) | -0.11 | (0.70) | -0.27 | (0.33) | 0.95 | | 0.01 | (0.97) | 0.05 | (0.85) | -0.30 | (0.28) |
| Corticospinal tract R | 0.69 | -0.05 | (0.86) | 0.09 | (0.76) | -0.04 | (0.87) | 0.49 | | -0.07 | (0.81) | 0.04 | (0.88) | -0.03 | (0.91) |
| Corticospinal tract L | 0.73 | 0.01 | (0.96) | 0.32 | (0.25) | -0.08 | (0.77) | 0.45 | | -0.26 | (0.35) | 0.14 | (0.62) | -0.22 | (0.44) |
| Medial lemniscus R | 0.73 | 0.26 | (0.35) | -0.13 | (0.65) | -0.01 | (0.98) | 0.61 | | 0.08 | (0.79) | -0.44 | (0.10) | 0.34 | (0.21) |
| Medial lemniscus L | 0.67 | 0.18 | (0.52) | -0.04 | (0.88) | 0.04 | (0.90) | 0.64 | | -0.02 | (0.95) | -0.56 | (0.03)* | 0.30 | (0.27) |
| Inferior cerebellar peduncle R | 0.57 | 0.30 | (0.28) | -0.11 | (0.69) | -0.25 | (0.38) | 0.62 | | 0.14 | (0.61) | -0.56 | (0.03)* | 0.34 | (0.22) |
| Inferior cerebellar peduncle L | 0.89 | 0.45 | (0.09) | -0.12 | (0.68) | -0.34 | (0.21) | 0.75 | | 0.06 | (0.83) | -0.63 | (0.01)* | 0.33 | (0.23) |
| Superior cerebellar peduncle R | 0.70 | 0.33 | (0.23) | 0.06 | (0.82) | -0.20 | (0.47) | 0.65 | | -0.12 | (0.66) | -0.30 | (0.28) | 0.19 | (0.49) |
| Superior cerebellar peduncle L | 0.75 | 0.13 | (0.64) | 0.16 | (0.58) | 0.07 | (0.80) | 0.74 | | -0.23 | (0.41) | -0.08 | (0.77) | 0.15 | (0.60) |
| Cerebral peduncle R | 0.54 | 0.13 | (0.65) | 0.31 | (0.26) | 0.02 | (0.96) | 0.09^++^ | | -0.42 | (0.11) | -0.23 | (0.42) | 0.20 | (0.47) |
| Cerebral peduncle L | 0.54 | 0.14 | (0.61) | 0.28 | (0.32) | -0.09 | (0.74) | 0.50 | | -0.23 | (0.41) | -0.07 | (0.81) | 0.20 | (0.48) |
| Anterior limb of internal capsule R | 0.94 | 0.55 | (0.03)* | -0.60 | (0.02)* | -0.60 | (0.02) | 0.81 | | 0.49 | (0.06) | -0.03 | (0.92) | -0.03 | (0.91) |
| Anterior limb of internal capsule L | 0.91 | 0.54 | (0.04)* | -0.61 | (0.02)* | -0.45 | (0.1) | 0.84 | | 0.45 | (0.09) | 0.30 | (0.28) | -0.26 | (0.36) |
| Posterior limb of internal capsule R | 0.78 | 0.44 | (0.10) | -0.46 | (0.08) | -0.42 | (0.11) | 0.84 | | 0.38 | (0.16) | 0.03 | (0.92) | -0.11 | (0.71) |
| Posterior limb of internal capsule L | 0.72 | 0.28 | (0.31) | -0.27 | (0.33) | 0.11 | (0.69) | 0.58 | | -0.08 | (0.79) | 0.31 | (0.26) | -0.25 | (0.36) |
| Retrolenticular part of internal capsule R | 0.88 | 0.36 | (0.19) | -0.22 | (0.42) | -0.40 | (0.14) | 0.81 | | 0.13 | (0.65) | -0.19 | (0.50) | -0.26 | (0.35) |
| Retrolenticular part of internal capsule L | 0.68 | 0.35 | (0.21) | -0.23 | (0.40) | -0.06 | (0.82) | 0.58 | | 0.03 | (0.91) | 0.23 | (0.42) | -0.21 | (0.46) |
| Anterior corona radiata R | 0.93 | 0.15 | (0.61) | -0.44 | (0.10) | -0.14 | (0.62) | 0.78 | | 0.20 | (0.47) | -0.24 | (0.4) | -0.19 | (0.51) |
| Anterior corona radiata L | 0.97 | 0.35 | (0.21) | -0.49 | (0.06) | -0.16 | (0.56) | 0.76 | | 0.32 | (0.25) | 0.24 | (0.39) | -0.41 | (0.13) |
| Superior corona radiata R | 0.97 | -0.24 | (0.39) | -0.52 | (0.05)* | 0.38 | (0.16) | 0.55 | | 0.15 | (0.59) | -0.32 | (0.24) | -0.34 | (0.22) |
| Superior corona radiata L | 0.93 | -0.13 | (0.65) | -0.41 | (0.12) | 0.15 | (0.6) | 0.50 | | 0.12 | (0.66) | 0.0 | (0.99) | -0.41 | (0.13) |
| Posterior corona radiata R | 0.96 | 0.15 | (0.60) | -0.28 | (0.32) | -0.27 | (0.32) | 0.84 | | 0.02 | (0.94) | -0.21 | (0.45) | -0.39 | (0.15) |
| Posterior corona radiata L | 0.87 | 0.25 | (0.38) | -0.16 | (0.56) | -0.20 | (0.48) | 0.89 | | 0.10 | (0.72) | 0.09 | (0.75) | -0.27 | (0.33) |
| Posterior thalamic radiation  (include optic radiation) R | 0.86 | 0.13 | (0.65) | 0.24 | (0.39) | -0.34 | (0.22) | 0.78 | | 0.19 | (0.51) | -0.28 | (0.31) | 0.31 | (0.26) |
| Posterior thalamic radiation  (include optic radiation) L | 0.78 | 0.30 | (0.27) | 0.23 | (0.41) | -0.14 | (0.62) | 0.85 | | -0.05 | (0.87) | 0.16 | (0.56) | -0.22 | (0.42) |
| Sagittal stratum R | 0.88 | 0.31 | (0.27) | -0.02 | (0.94) | -0.63 | (0.01)* | 0.79 | | 0.08 | (0.79) | -0.22 | (0.42) | -0.11 | (0.71) |
| Sagittal stratum L | 0.79 | 0.27 | (0.34) | -0.29 | (0.29) | -0.07 | (0.80) | 0.86 | | 0.43 | (0.11) | 0.16 | (0.56) | -0.21 | (0.45) |
| External capsule R | 0.93 | 0.15 | (0.58) | -0.54 | (0.04)* | -0.12 | (0.67) | 0.65 | | 0.29 | (0.29) | 0.21 | (0.46) | -0.15 | (0.61) |
| External capsule L | 0.90 | 0.21 | (0.45) | -0.31 | (0.26) | 0.0 | (0.99) | 0.58 | | 0.13 | (0.64) | 0.39 | (0.15) | -0.42 | (0.12) |
| Cingulum (cingulate gyrus) R | 0.92 | 0.27 | (0.33) | -0.41 | (0.13) | -0.13 | (0.63) | 0.74 | | 0.22 | (0.44) | 0.17 | (0.56) | -0.26 | (0.35) |
| Cingulum (cingulate gyrus) L | 0.92 | 0.18 | (0.52) | -0.36 | (0.18) | -0.03 | (0.93) | 0.86 | | 0.22 | (0.43) | 0.55 | (0.03)* | -0.29 | (0.30) |
| Cingulum (hippocampus) R | 0.85 | 0.35 | (0.19) | 0.10 | (0.73) | -0.29 | (0.3) | 0.81 | | -0.17 | (0.55) | -0.46 | (0.09) | -0.15 | (0.60) |
| Cingulum (hippocampus) L | 0.80 | 0.07 | (0.80) | 0.32 | (0.24) | -0.05 | (0.86) | 0.77 | | -0.27 | (0.33) | -0.31 | (0.25) | 0.15 | (0.60) |
| Fornix (cres) / Stria terminalis R | 0.93 | 0.34 | (0.21) | -0.01 | (0.96) | -0.29 | (0.29) | 0.79 | | 0.14 | (0.61) | -0.39 | (0.15) | 0.31 | (0.27) |
| Fornix (cres) / Stria terminalis L | 0.67 | 0.32 | (0.24) | -0.11 | (0.70) | -0.02 | (0.94) | 0.91 | | 0.06 | (0.82) | 0.41 | (0.13) | -0.18 | (0.52) |
| Superior longitudinal fasciculus R | 0.85 | 0.51 | (0.05)* | -0.57 | (0.03)* | -0.56 | (0.03)* | 0.82 | | 0.46 | (0.09) | 0.19 | (0.49) | -0.44 | (0.10) |
| Superior longitudinal fasciculus L | 0.80 | 0.40 | (0.14) | -0.32 | (0.25) | -0.08 | (0.76) | 0.81 | | 0.03 | (0.90) | 0.42 | (0.11) | -0.32 | (0.24) |
| Superior fronto-occipital fasciculus R | 0.81 | -0.39 | (0.15) | -0.02 | (0.93) | 0.26 | (0.35) | 0.45 | | -0.12 | (0.66) | -0.02 | (0.96) | 0.12 | (0.67) |
| Superior fronto-occipital fasciculus L | 0.74 | 0.31 | (0.27) | -0.30 | (0.28) | -0.03 | (0.91) | 0.41 | | -0.04 | (0.89) | 0.47 | (0.08) | -0.48 | (0.07) |
| Uncinate fasciculus R | 0.80 | 0.01 | (0.97) | -0.06 | (0.84) | 0.13 | (0.65) | 0.69 | | -0.24 | (0.39) | 0.24 | (0.39) | -0.32 | (0.24) |
| Uncinate fasciculus L | 0.91 | 0.03 | (0.93) | -0.56 | (0.03)* | -0.15 | (0.59) | 0.89 | | 0.39 | (0.15) | -0.02 | (0.95) | -0.02 | (0.94) |
| Tapetum R | 0.95 | 0.14 | (0.62) | 0.19 | (0.50) | -0.17 | (0.54) | 0.85 | | -0.30 | (0.28) | -0.26 | (0.34) | 0.06 | (0.82) |
| Tapetum L | 0.87 | 0.11 | (0.71) | 0.47 | (0.08) | 0.18 | (0.53) | 0.83 | | -0.18 | (0.53) | 0.03 | (0.93) | -0.15 | (0.60) |

Abbreviations: ICC = intraclass correlation coefficient; * = significant (p>0.05); **^++^** = minimum for FA or MD.

Table S3. Test-retest reliability of functional connectivity estimates for between regions within cortical resting-state networks.

| **Connection within DMN** | **Mean FC-Z (SD) test** | **Mean FC-Z (SD) retest** | **Mean FC-Z (SD)**  **change** | **ICC [95% CI]** |
| --- | --- | --- | --- | --- |
| MPFC – LLP | +0.55 (0.20) | +0.53 (0.14) | –0.02 (0.23) | 0.16 [–0.37 to 0.61] |
| MPFC – RLP | +0.56 (0.22) | +0.53 (0.24) | –0.04 (0.28) | 0.27 [–0.26 to 0.68] |
| MPFC – PCC | +0.49 (0.26) | +0.53 (0.19) | +0.05 (0.23) | 0.48 [–0.02 to 0.79] |
| LLP – RLP | +0.68 (0.19) | +0.73 (0.22) | +0.10 (0.17) | 0.65 [0.23 to 0.87] |
| LLP – PCC | +0.57 (0.18) | +0.57 (0.22) | –0.01 (0.23) | 0.34 [–0.19 to 0.72] |
| RLP – PCC | +0.59 (0.25) | +0.59 (0.29) | –0.01 (0.22) | 0.69 [0.29 to 0.88] |
| **Connection within SMN** | **Mean FC-Z (SD) test** | **Mean FC-Z (SD) retest** | **Mean FC-Z (SD)**  **change** | **ICC [95% CI]** |
| Left lateral – Right lateral | +0.90 (0.30) | +0.88 (0.24) | –0.09 (0.32) | 0.31 [–0.22 to 0.70] |
| Left lateral – Superior | +0.67 (0.21) | +0.63 (0.25) | –0.06 (0.25) | 0.39 [–0.13 to 0.74] |
| Right lateral – Superior | +0.64 (0.24) | +0.63 (0.23) | –0.03 (0.25) | 0.45 [–0.07 to 0.77] |
| **Connection within VN** | **Mean FC-Z (SD) test** | **Mean FC-Z (SD) retest** | **Mean FC-Z (SD)**  **change** | **ICC [95% CI]** |
| Medial – Occipital | +0.44 (0.29) | +0.58 (0.31) | +0.19 (0.30) | 0.50 [0.01 to 0.80] |
| Medial – Left lateral | +0.62 (0.20) | +0.57 (0.29) | –0.08 (0.21) | 0.66 [0.23 to 0.87] |
| Medial – Right lateral | +0.61 (0.20) | +0.50 (0.25) | –0.15 (0.25) | 0.41 [–0.11 to 0.75] |
| Occipital – Left lateral | +0.50 (0.22) | +0.63 (0.32) | +0.19 (0.30) | 0.41 [–0.11 to 0.75] |
| Occipital – Right lateral | +0.51 (0.21) | +0.61 (0.26) | +0.14 (0.26) | 0.39 [–0.13 to 0.74] |
| Left lateral – Right lateral | +0.91 (0.26) | +0.89 (0.21) | –0.10 (0.28) | 0.31 [–0.22 to 0.70] |
| **Connection within SN** | **Mean FC-Z (SD) test** | **Mean FC-Z (SD) retest** | **Mean FC-Z (SD)**  **change** | **ICC [95% CI]** |
| ACC – Left anterior insula | +0.59 (0.20) | +0.60 (0.21) | +0.01 (0.26) | 0.19 [–0.34 to 0.63] |
| ACC – Right anterior insula | +0.58 (0.16) | +0.58 (0.21) | +0.01 (0.22) | 0.29 [–0.24 to 0.69] |
| ACC – Left RPFC | +0.52 (0.19) | +0.43 (0.18) | –0.12 (0.14) | 0.72 [0.35 to 0.90] |
| ACC – Right RPFC | +0.47 (0.15) | +0.35 (0.21) | –0.14 (0.20) | 0.42 [–0.10 to 0.76] |
| ACC – Left SMG | +0.31 (0.26) | +0.30 (0.19) | –0.02 (0.21) | 0.56 [0.08 to 0.83] |
| ACC – Right SMG | +0.31 (0.18) | +0.29 (0.20) | –0.03 (0.23) | 0.27 [–0.26 to 0.68] |
| Left anterior insula – Right anterior insula | +0.77 (0.21) | +0.73 (0.23) | –0.08 (0.20) | 0.56 [0.09 to 0.83] |
| Left anterior insula – Left RPFC | +0.51 (0.14) | +0.40 (0.24) | –0.13 (0.18) | 0.59 [0.13 to 0.84] |
| Left anterior insula – Right RPFC | +0.35 (0.24) | +0.22 (0.25) | –0.14 (0.24) | 0.51 [0.02 to 0.80] |
| Left anterior insula – Left SMG | +0.58 (0.27) | +0.54 (0.26) | –0.06 (0.35) | 0.13 [–0.40 to 0.59] |
| Left anterior insula – Right SMG | +0.53 (0.20) | +0.47 (0.21) | –0.08 (0.24) | 0.33 [–0.20 to 0.71] |
| Right anterior insula – Left RPFC | +0.48 (0.25) | +0.38 (0.28) | –0.13 (0.22) | 0.68 [0.27 to 0.88] |
| Right anterior insula – Right RPFC | +0.47 (0.29) | +0.36 (0.27) | –0.13 (0.19) | 0.78 [0.45 to 0.92] |
| Right anterior insula – Left SMG | +0.53 (0.20) | +0.46 (0.26) | –0.09 (0.27) | 0.32 [–0.21 to 0.70] |
| Right anterior insula – Right SMG | +0.65 (0.23) | +0.60 (0.25) | –0.08 (0.22) | 0.59 [0.13 to 0.84] |
| Left RPFC – Right RPFC | +0.68 (0.25) | +0.58 (0.22) | –0.18 (0.24) | 0.47 [–0.04 to 0.78] |
| Left RPFC – Left SMG | +0.36 (0.15) | +0.31 (0.23) | –0.05 (0.20) | 0.45 [–0.06 to 0.78] |
| Left RPFC – Right SMG | +0.36 (0.24) | +0.29 (0.26) | –0.08 (0.19) | 0.70 [0.32 to 0.89] |
| Right RPFC – Left SMG | +0.21 (0.29) | +0.11 (0.25) | –0.11 (0.34) | 0.15 [–0.38 to 0.60] |
| Right RPFC – Right SMG | +0.30 (0.34) | +0.23 (0.35) | –0.07 (0.25) | 0.75 [0.41 to 0.91] |
| Left SMG – Right SMG | +0.65 (0.26) | +0.56 (0.27) | –0.13 (0.27) | 0.48 [–0.03 to 0.79] |
| **Connection within DAN** | **Mean FC-Z (SD) test** | **Mean FC-Z (SD) retest** | **Mean FC-Z (SD)**  **change** | **ICC [95% CI]** |
| Left FEF – Right FEF | +0.45 (0.17) | +0.45 (0.24) | –0.00 (0.30) | –0.06^++^ [–0.54 to 0.45] |
| Left FEF – Left IPS | +0.28 (0.24) | +0.45 (0.27) | +0.19 (0.20) | 0.70 [0.31 to 0.89] |
| Left FEF – Right IPS | +0.20 (0.16) | +0.30 (0.25) | +0.11 (0.19) | 0.59 [0.13 to 0.84] |
| Right FEF – Left IPS | +0.22 (0.20) | +0.25 (0.22) | +0.03 (0.26) | 0.24 [–0.29 to 0.66] |
| Right FEF – Right IPS | +0.29 (0.29) | +0.25 (0.20) | –0.04 (0.27) | 0.42 [–0.10 to 0.76] |
| Left IPS – Right IPS | +0.69 (0.16) | +0.66 (0.19) | –0.05 (0.19) | 0.38 [–0.15 to 0.74] |
| **Connection within FPN** | **Mean FC-Z (SD) test** | **Mean FC-Z (SD) retest** | **Mean FC-Z (SD)**  **change** | **ICC [95% CI]3,1 FC-Z (95% CI)** |
| Left LPFC – Left PPC | +0.55 (0.26) | +0.59 (0.24) | +0.05 (0.19) | 0.72 [0.34 to 0.90] |
| Left LPFC – Right LPFC | +0.54 (0.23) | +0.58 (0.19) | +0.06 (0.30) | –0.03 [–0.52 to 0.47] |
| Left LPFC – Right PPC | +0.31 (0.21) | +0.36 (0.25) | +0.05 (0.22) | 0.56 [0.09 to 0.83] |
| Left PPC – Right LPFC | +0.41 (0.18) | +0.49 (0.14) | +0.10 (0.19) | 0.26 [–0.28 to 0.67] |
| Left PPC – Right PPC | +0.65 (0.21) | +0.69 (0.19) | +0.08 (0.24) | 0.28 [–0.25 to 0.68] |
| Right LPFC – Right PPC | +0.61 (0.21) | +0.67 (0.22) | +0.10 (0.31) | –0.02 [–0.51 to 0.48] |
| **Connection within LN** | **Mean FC-Z (SD) test** | **Mean FC-Z (SD) retest** | **Mean FC-Z (SD)**  **change** | **ICC [95% CI]** |
| Left IFG – Right IFG | +0.61 (0.31) | +0.55 (0.23) | –0.08 (0.24) | 0.61 [0.16 to 0.85] |
| Left IFG – Left pSTG | +0.68 (0.29) | +0.58 (0.21) | –0.16 (0.30) | 0.31 [–0.22 to 0.70] |
| Left IFG – Right pSTG | +0.49 (0.27) | +0.37 (0.20) | –0.14 (0.19) | 0.70 [0.30 to 0.89] |
| Right IFG – Left pSTG | +0.49 (0.22) | +0.40 (0.20) | –0.11 (0.23) | 0.39 [–0.14 to 0.74] |
| Right IFG – Right pSTG | +0.58 (0.17) | +0.51 (0.24) | –0.11 (0.21) | 0.48 [–0.03 to 0.79] |
| Left pSTG –Right pSTG | +0.72 (0.27) | +0.72 (0.27) | –0.01 (0.22) | 0.67 [0.26 to 0.88] |
| **Connection within CBN** | **Mean FC-Z (SD) test** | **Mean FC-Z (SD) retest** | **Mean FC-Z (SD)**  **change** | **ICC [95% CI]** |
| Anterior –Posterior | +0.57 (0.26) | +0.54 (0.12) | –0.05 (0.28) | –0.01 [–0.50 to 0.49] |

Abbreviations: FC-Z = r-to-Z-transformed functional connectivity; SD = standard deviation; ICC = intraclass correlation coefficient; CI = confidence interval; **^++^** = lowest ICC; DMN = default mode network; SMN = sensorimotor network; VN = visual network; SN = salience network; DAN = dorsal attention network; FPN = frontoparietal network; LN = language network; CBN = cerebellar network.

Table S4. Test-retest reliability of average functional connectivity estimates for connections between cortical resting-state networks.

| **Connection between RSNs** | **Mean FC-Z (SD)**  **test** | **Mean FC-Z (SD)**  **retest** | **Mean FC-Z (SD)**  **change** | **ICC [95% CI]** |
| --- | --- | --- | --- | --- |
| Default mode – Sensorimotor | –0.03 (0.19) | –0.07 (0.26) | –0.04 (0.29) | –0.03 [–0.52 to 0.48] |
| Default mode – Visual | +0.29 (0.20) | +0.31 (0.24) | +0.02 (0.26) | +0.49 [–0.01 to 0.79] |
| Default mode – Salience | –0.06 (0.29) | –0.00 (0.30) | +0.05 (0.26) | +0.71 [0.33 to 0.89] |
| Default mode – Dorsal attention | –0.10 (0.21) | –0.16 (0.22) | –0.06 (0.22) | +0.25 [–0.28 to 0.66] |
| Default mode – Frontoparietal | +0.26 (0.13) | +0.35 (0.24) | +0.10 (0.29) | +0.05 [–0.46 to 0.53] |
| Default mode – Language | +0.14 (0.21) | +0.22 (0.23) | +0.09 (0.33) | +0.01 [–0.49 to 0.51] |
| Default mode – Cerebellum | +0.34 (0.20) | +0.40 (0.21) | +0.06 (0.27) | –0.10 [–0.57 to 0.42] |
| Sensorimotor – Visual | +0.18 (0.18) | +0.19 (0.26) | +0.01 (0.26) | +0.09 [–0.18 to 0.72] |
| Sensorimotor – Salience | +0.19 (0.15) | +0.17 (0.20) | –0.02 (0.25) | +0.08 [–0.44 to 0.55] |
| Sensorimotor – Dorsal attention | +0.45 (0.17) | +0.36 (0.19) | –0.11 (0.18) | +0.23 [–0.30 to 0.65] |
| Sensorimotor – Frontoparietal | –0.17 (0.13) | –0.32 (0.25) | –0.16 (0.25) | –0.02 [–0.51 to 0.48] |
| Sensorimotor – Language | +0.19 (0.21) | +0.16 (0.16) | –0.04 (0.23) | +0.26 [–0.27 to 0.67] |
| Sensorimotor – Cerebellum | +0.06 (0.21) | –0.03 (0.25) | –0.10 (0.27) | +0.43 [–0.09 to 0.76] |
| Visual – Salience | +0.10 (0.19) | +0.23 (0.19) | +0.13 (0.22) | +0.32 [–0.21 to 0.71] |
| Visual – Dorsal attention | +0.15 (0.19) | –0.00 (0.22) | –0.15 (0.24) | +0.26 [–0.27 to 0.67] |
| Visual – Frontoparietal | –0.08 (0.20) | –0.10 (0.18) | –0.02 (0.16) | +0.57 [0.11 to 0.83] |
| Visual – Language | +0.06 (0.16) | +0.15 (0.20) | +0.09 (0.17) | +0.63 [0.19 to 0.86] |
| Visual – Cerebellum | +0.20 (0.21) | +0.09 (0.21) | –0.11 (0.22) | +0.21 [–0.32 to 0.64] |
| Salience – Dorsal attention | +0.26 (0.23) | +0.14 (0.20) | –0.13 (0.31) | +0.08 [–0.44 to 0.55] |
| Salience – Frontoparietal | +0.06 (0.29) | +0.06 (0.27) | –0.01 (0.20) | +0.72 [0.35 to 0.90] |
| Salience – Language | +0.38 (0.19) | +0.31 (0.21) | –0.07 (0.23) | +0.53 [0.04 to 0.81] |
| Salience – Cerebellum | +0.15 (0.24) | +0.07 (0.19) | –0.08 (0.24) | +0.46 [–0.05 to 0.78] |
| Dorsal attention – Frontoparietal | +0.12 (0.21) | +0.04 (0.27) | –0.08 (0.25) | +0.49 [–0.02 to 0.79] |
| Dorsal attention – Language | –0.01 (0.27) | –0.01 (0.21) | –0.01 (0.22) | +0.53 [0.05 to 0.81] |
| Dorsal attention – Cerebellum | +0.07 (0.25) | +0.03 (0.27) | –0.04 (0.30) | +0.34 [–0.19 to 0.72] |
| Frontoparietal – Language | +0.30 (0.25) | +0.27 (0.22) | –0.03 (0.23) | +0.45 [–0.06 to 0.78] |
| Frontoparietal – Cerebellum | +0.47 (0.12) | +0.52 (0.20) | +0.07 (0.21) | –0.13^++^ [–0.59 to 0.39] |
| Language – Cerebellum | +0.41 (0.20) | +0.33 (0.14) | –0.09 (0.22) | +0.14 [–0.39 to 0.59] |

Abbreviations: FC-Z = r-to-Z-transformed functional connectivity; SD = standard deviation; ICC = intraclass correlation coefficient; CI = confidence interval; **^++^** = lowest ICC.


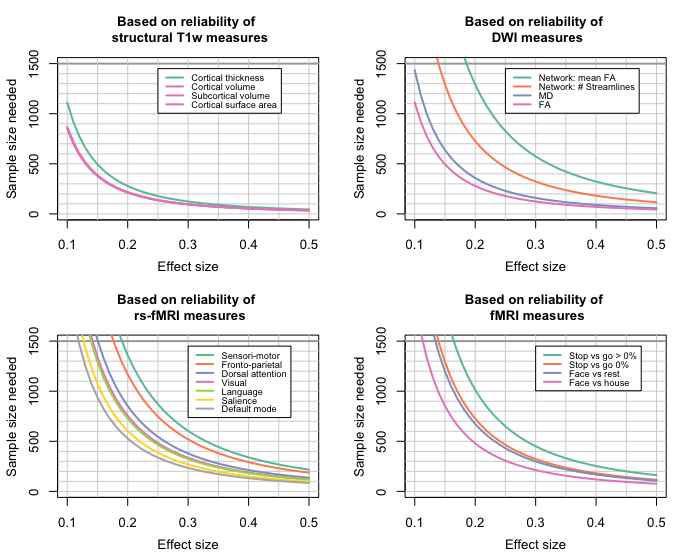


**Figure S2. Sample size estimations for each type of scan based on the ICCs.** The sample size (y-axis), needed to detect an effect of interest with 80% power (beta=0.2 and alpha=0.05), is modelled for different effect sizes (Cohen’s D, x-axis). For each type of scan the test-retest ICCs are used to estimate the amount of error. All legends are ordered from lowest ICC to higher ICCs. *Top left,* for the structural T1-weighted scans the average of the regional ICCs is used for cortical thickness, cortical volume, subcortical volume and cortical surface area (ICCs lowest to highest: 0.84, 0.95, 0.96 and 0.98). Please note that the last three lines are nearly identical and therefore overlap in the plot. *Top right,* for the diffusion-weighted images the ICCs correspond to the average FA and number of streamlines over all edges and the average regional ICC of the MD and FA values (ICCs lowest to highest: 0.39, 0.52, 0.74 and 0.84). *Bottom left,* for the resting-state fMRI scans the ICCs used for the sample size estimation correspond to the reliability of the functional connectivity within cerebral cortical resting-state networks (ICCs lowest to highest: 0.38, 0.41, 0.48, 0.51, 0.52, 0.57 and 0.61). *Bottom right,* for the task-based fMRI the average regional ICCs used for the sample size estimation correspond to two contrasts for the inhibition task and two contrasts for the emotion task indicated in the legend (ICCs lowest to highest: 0.44, 0.52, 0.54 and 0.64).
